# Discovery of microRNA-like RNAs during early fruiting body development in the model mushroom *Coprinopsis cinerea*

**DOI:** 10.1371/journal.pone.0198234

**Published:** 2018-09-19

**Authors:** Amy Yuet Ting Lau, Xuanjin Cheng, Chi Keung Cheng, Wenyan Nong, Man Kit Cheung, Raymond Hon-Fu Chan, Jerome Ho Lam Hui, Hoi Shan Kwan

**Affiliations:** 1 School of Life Sciences, The Chinese University of Hong Kong, Shatin, New Territories, Hong Kong; 2 Department of Mathematics, The Chinese University of Hong Kong, Shatin, New Territories, Hong Kong; 3 Simon F.S. Li Marine Science Laboratory of School of Life Sciences and Centre of Soybean of State Key Laboratory of Agrobiotechnology, The Chinese University of Hong Kong, Shatin, New Territories, Hong Kong; Friedrich Schiller University, GERMANY

## Abstract

*Coprinopsis cinerea* is a model mushroom particularly suited for the study of fungal fruiting body development and the evolution of multicellularity in fungi. While microRNAs (miRNAs) have been extensively studied in animals and plants for their essential roles in post-transcriptional regulation of gene expression, miRNAs in fungi are less well characterized and their potential roles in controlling mushroom development remain unknown. To identify miRNA-like RNAs (milRNAs) in *C*. *cinerea* and explore their expression patterns during the early developmental transition of mushroom development, small RNA libraries of vegetative mycelium and primordium were generated and putative milRNA candidates were identified following the standards of miRNA prediction in animals and plants. Two out of 22 novel predicted milRNAs, cci-milR-12c and cci-milR-13e-5p, were validated by northern blot and stem-loop reverse transcription real-time PCR. Cci-milR-12c was differentially expressed whereas the expression levels of cci-milR-13e-5p were similar in the two developmental stages. Target prediction of the validated milRNAs resulted in genes associated with fruiting body development, including pheromone, hydrophobin, cytochrome P450, and protein kinase. Essential genes for miRNA biogenesis, including three coding for Dicer-like (DCL), one for Argonaute (AGO), one for AGO-like and one for quelling deficient-2 (QDE-2) proteins, were also identified in the *C*. *cinerea* genome. Phylogenetic analysis showed that the DCL and AGO proteins of *C*. *cinerea* were more closely related to those in other basidiomycetes and ascomycetes than to those in animals and plants. Taken together, our findings provided the first evidence for milRNAs in the model mushroom and their potential roles in regulating fruiting body development. New information on the evolutionary relationship of milRNA biogenesis proteins across kingdoms has also provided new insights for guiding further functional and evolutionary studies of miRNAs.

## Introduction

Small non-coding RNAs (sRNAs), about 20–30 nucleotides (nt) in length, are the regulators of RNA interference (RNAi), a conserved eukaryotic gene silencing mechanism [1]. sRNAs are categorized into three groups based on their origin and functions: small interfering RNAs (siRNAs), piwi-interacting RNAs (piRNAs) and microRNAs (miRNAs) [2]. In fungi, RNAi-related machineries are mainly responsible for genomic defence, heterochromatin formation and gene regulation [3]. For example, siRNAs mediate quelling and meiotic silencing of unpaired DNA as genomic surveillance against viral infection in *Cryphpnectria parasitica*, and against transposon invasion and silencing unpaired DNA in *Neurospora crassa* [3, 4, 5]. Most of the descriptions of RNAi pathways of sRNAs in various fungi have focused only on the siRNA-directed pathways.

MiRNAs are present in nearly all eukaryotic lineages. They play essential roles in various biological processes by mediating post-transcriptional gene silencing to regulate gene expression through base pairing their seed region (2–7 nt at the 5’-end) to the untranslated region (UTR) or opening reading frame of their target genes [6–13]. In plants, miRNAs play roles in tissue morphogenesis, stress response and stem development through mRNA cleavage after perfect complementarity binding to their targets [12]. In animals, miRNAs regulate cell proliferation and differentiation, apoptosis, and different metabolic pathways during developmental transition by miRNA-mediated translational repression [6, 7, 8, 9, 11, 14]. The first miRNA-like RNA (milRNA) in filamentous fungi was described in *N*. *crassa* only in 2010, more than a decade later than in animals and plants [15]. Although milRNAs have been subsequently discovered in other fungi, such as *Sclerotinia sclerotiorum*, *Trichoderma reesei*, *Penicillium marneffei*, *Fusarium oxysporum*, and *Antrodia cinnamonmea*, the potential roles of milRNAs in the developmental processes of mushroom forming fungi are still largely unknown [16– 20].

Fungi, ranging from the simplest unicellular yeasts to macroscopic mushrooms, possess a fascinating morphological diversity [21]. *Coprinopsis cinerea*, commonly known as the ink cap, is one of the most morphologically complex fungi and has a well-characterized genome [22]. *C*. *cinerea* is also a model mushroom that is commonly used to study the developmental processes in higher basidiomycete fungi. Under nutrient depletion and normal day-night rhythm, the undifferentiated vegetative mycelium of *C*. *cinerea* undergoes dynamic genetic and physiological changes to form a multihyphal structure, known as the fruiting body, through hyphal aggregation and mycelial differentiation [23–25]. Fruiting body development of *C*. *cinerea* is a rapid but complex process, consisting of six main stages: mycelium, initials, stage 1 and 2 primordium, young fruiting body, and mature fruiting body [22]. The entire process can be completed within two weeks when the fungus is cultured on artificial media with optimal conditions [26,27]. Understanding the molecular regulatory mechanisms during fruiting body initiation and development is one of the major goals of mycological studies. The most significant transcriptomic switch has been shown to occur during the transition from mycelium to primordium, which represents a developmental transition from a loose, undifferentiated structure to a compact and well-organized multicellular body plan [28, 29].

The regulatory roles of miRNAs have been demonstrated in various multicellular organisms. In fungi, the key components of RNA regulatory networks and stage-specific milRNAs have also been reported [11, 15, 20, 30–35]. Some miRNAs in animals and plants are expressed in a stage-specific or tissue-specific manner, suggesting their potential roles in maintaining tissue specificity and functions [11, 36–39]. Here, we hypothesized that miRNAs also regulate developmental transition in the mushroom forming fungus *C*. *cinerea*. Prediction and identification of milRNAs and their targets in *C*. *cinerea* are feasible based on the published genome sequence data and transcriptomic profiles of the early developmental transition in *C*. *cinerea* [22, 28, 29]. In this study, we used high-throughput small RNA (sRNA) sequencing to computationally identify 22 putative milRNA candidates in the mycelium and primordium stages of *C*. *cinerea*. Two milRNAs, namely cci-milR-12c and cci-milR-13e-5p, were validated using northern blot analysis, and their expression levels were examined by stem-loop RT-qPCR in both developmental stages. One of the milRNA candidates, cci-milR-12c, was found to be differentially expressed. Genes encoding putative Dicer-like (DCL), Argonuate (AGO), AGO-like and quelling deficient-2 (QDE-2) proteins were identified in the *C*. *cinerea* genome [22]. Our results have provided evidence for the presence of milRNAs in *C*. *cinerea*, revealed their potential targets, and demonstrated the differential expression of milRNAs during the early developmental stages of the model mushroom. Our study has facilitated the understanding of the diversified regulatory roles of milRNAs and the molecular mechanisms of fruiting body development in higher basidiomycete fungi.

## Materials and methods

### *C*. *cinerea* strains and growth conditions

The *C*. *cinerea* strain used for the identification of milRNAs and core milRNA biogenesis proteins is a dikaryon, mated from the monokaryotic strains J6;5–4 and J6;5–5 [40]. Two monokaryons were generated from single spore isolates of a dikaryon that had been backcrossed with the reference strain Okayama 7#130 for five generations. The homokaryotic fruiting strain #326 *(A43mut B43mut pab1-1)* was also used in the siRNA-mediated Dicer knockdown analysis [41]. The strains were cultured on YMD medium containing 0.4% yeast extract, 1% malt extract, and 0.4% glucose with Bacto agar. Mycelia were cultivated on agar plates at 37 °C for about 4–5 days until the mycelium grew over the whole agar surface and reached the edge of plates. Fruiting body formation was induced by incubating the mycelium culture at 25 °C under a light/dark regime of 12/12 h [22,42,43]. The incubator was kept at a relative humidity > 60% for the production of fruiting bodies.

### RNA isolation and sRNA sequencing

Samples were collected from two biological replicates for each developmental stage of *C*. *cinerea*. In brief, total RNA was extracted from mycelium (MYC) (4–5 days in the dark) and primodium (PRI) (~ 6–7 mm tall, 3 days in the light) using mirVana miRNA Isolation Kit (Ambion) and treated with TURBO DNA-free Kit (Ambion) in accordance with the manufacturer’s instructions. Mycelia from four agar plates and 4–5 independent primordium structures were harvested and pooled to form one replicate. All samples were stored at −80 °C. The concentration and quality of RNA samples were checked using an Agilent 2100 Bioanalyzer. Using total RNA as the starting material, sRNA sequencing was performed by Macrogen (Korea) on a Hiseq 2500 platform (Illumina). The sRNA sequence dataset was deposited to Sequence Read Archive (SRA) of National Center for Biotechnology Information (NCBI) under the accession no. SRP150974.

### Bioinformatics analysis of sRNAs and prediction of milRNA candidates

Raw sequence reads were filtered to remove low quality reads with a Phred score lower than 20, adaptor and primer sequences, and reads shorter than 18 nt (Macrogen, Seoul, Korea). High quality reads were then used to build a non-redundant dataset in which reads identical in length and identity were clustered (Macrogen, Seoul, Korea). Clean unique reads were searched on Rfam v.9.1 to identify other types of small ncRNAs, such as rRNA, tRNA and snRNA (Macrogen, Seoul, Korea) [44]. Since previous studies have identified miRNAs derived from other small ncRNAs, clean clustered reads with 18–30 nt, including those aligned to tRNAs and rRNAs, were subsequently mapped to the *C*. *cinerea* genome (NCBI assembly accession: AACS00000000) using Bowtie and only perfectly matched sRNA reads were selected for milRNA prediction (Macrogen, Seoul, Korea) [45].

For milRNA candidate prediction, short clean reads ranging from 18–30 nt were first aligned to miRBase v.21 to categorize known miRNAs [46] and were clustered together by 95% similarity using CD-HIT [47]. An in-house Perl program was then developed to identify the novel miRNAs. Since *N*. *crassa* demonstrated a wider range of precursor length than that in plants, the remaining mapped sRNA sequences were first extended on the genome to 51–150 bp in length to form a precursor-like hairpin structure [15]. Secondary structures of the extended sequence were computed by RNAfold in Vienna RNA package 2.0 with GU wobble base pair allowed. Putative milRNA candidates were selected using the following criteria: (1) sRNA that formed a hairpin structure with minimum free energy (MFE) of folding ≤ -20 kcal/mol; (2) the predicted region contained at least 18 bp and the sRNA resided in this stem region; (3) only one loop with at least 4 bp was present in the predicted hairpin; (4) a RANDFOLD p-value of the predicted secondary structures < 0.01; and (3) with at least four reads [48,49,50]. The Perl script of miRNA prediction was deposited on figshare at https://figshare.com/s/10aa5707773d496e2c15.

### Validation of milRNAs by northern blot analysis

Northern blot analysis of milRNA identification was performed according to the protocol of Kim et al. with double-labeled digoxigenin (DIG) oligonucleotide probes instead of locked nucleic acids (LNA) probes [51]. Briefly, total RNA samples (5–15 ug) from the two different developmental stages were resolved on a 15% denaturing polyacrylamide gel with 8M Urea in 1X TBE. The RNA gels were then transferred to Hybond-N+ (Amersham Biosciences) at 10–15 V (30–60 min) using a Trans-Blot SD semi-dry transfer cell (Bio-Rad). Cross-linking, hybridization and membrane detection were performed as previously described [51]. Cross-linking was performed using freshly prepared 1-ethyl-3-(3-dimethylaminopropyl) carbodiimide (EDC) reagent at 60 °C for 1 hr. Membranes were hybridized overnight in ULTRAhyb^™^ hybridization buffer (Ambion) with specific double DIG-labeled oligonucleotide probes synthesized by Integrated DNA Technologies at 37 °C. Sequences of the probes used against the putative milRNAs were as follows: ccin-milR-12c, 5’-AAAGGTAGTGGTATTTCAACGGCGCC-3’; ccin-milR-13e-5p, 5’-AGTCCCTACTAGGTCCCGAG-3’. Probe detection was performed using DIG luminescent detection kit in accordance with the manufacturer’s instructions (Roche) and photoemissions were detected using the ChemiDoc-It Imaging System (Bio-rad).

### Identification and phylogenetic analysis of DCL and AGO protein genes

One AGO (XP_001837237.2), one AGO-like protein (XP_001837864.2) and a QDE-2 protein (XP_001838344.1) were found in the annotated protein sequences of *C*. *cinerea* from *GenBank* (AACS00000000) [22]. For the phylogenetic analyses of the two main effector proteins in miRNA biogenesis, Dicer and AGO, corresponding protein sequences of animals, plants and some ascomycete fungi were downloaded from UniProt (http://www.uniprot.org/). Based on the annotated protein sequences of *N*. *crassa* DCL-1, DCL-2 (XP_961898.1, XP_963538.3) and QDE-2 (XP_958586.1), three DCL proteins of *C*. *cinerea* (XP_002911949.1, XP_001837094.2, XP_001840952.1), and some ascomycete and basidiomycete fungi were identified using BLASTP against the JGI database (https://genome.jgi.doe.gov/) [15]. An E-value of ≤ 10E-10 and an identity ≥ 25% were used as the cutoffs in the BLASTP searches. The functional domains of the corresponding proteins in *C*. *cinerea* were predicted using Pfam and SMART [52, 53]. Phylogenetic trees of the proteins were constructed by the maximum likelihood method with 1000 bootstrap replicates using MEGA 7 [54].

### Experimental quantification of milRNAs and biogenesis proteins by RT-qPCR

The sequence-specific TaqMan MicroRNA Assays and TaqMan small RNA Assays (Life Technologies) were used for RT-qPCR of cci-milR-12c and cci-milR-13e-5p, and the 5S rRNA (endogenous control), respectively. Reverse transcription was performed using TaqMan MicroRNA Reverse Transcription Kit (Applied Biosystems, Inc). Results from the 5S rRNA were used for normalization. cDNA was amplified in 20 uL reaction mixtures containing TaqMan Universal PCR Master Mix, no AmpErase UNG (Applied Biosystem) using standard qPCR conditions (95 °C for 10 min, followed by 40 cycles of 95 °C for 15 sec and 60 °C for 1 min) [55].

To examine the expression levels of the Dicer and AGO proteins in *C*. *cinerea*, total RNA was reverse transcribed to cDNA using Transcriptor First Strand cDNA Synthesis Kit (Roche Applied Science) with random hexamer primers. Real-time PCR analysis was performed using the SsoAdvanced Universal SYBR Green Supermix (Bio-rad), with 1 μl of 10 μM gene-specific forward and reverse primers (S1 Table). Thermal cycling was performed for 35–40 cycles. Each cycle consisted of polymerase activation at 95 °C for 30 sec, denaturation at 95 °C for 5–15 sec, and extension at 60 °C for 1 min. The relative expressions of DCL, AGO, AGO-like and QDE-2 proteins were normalized against 18S rRNA with forward primer (5′-GCCTGTTTGAGTGTCATTAAATTCTC-3′) and reverse primer (5′-CTGCAACCCCCACATCCA-3′). All the cycling reactions were performed in triplicate and the cycle threshold fluorescence data were recorded on an ABI 7500 Fast Real-Time PCR system (Applied Biosystems). The comparative Ct method (ΔΔCt) was exploited to calculate the relative expression levels of both validated milRNAs, DCLs, AGO, AGO-like and QDE-2 proteins. Statistical analysis was performed by Student’s t-tests. A P-value <0.05 was considered statistically significant.

### Dicer-like proteins knockdown mediated by siRNAs

At least two sequence-specific siRNA targeting separate regions for each DCL mRNA were transfected into the stipe of primordium twice using needle and syringe to enhance the efficiency and effectiveness of knockdown [56]. The 5’-3’ sequences of the sense and antisense strands of synthetic Stealth siRNA duplexes (Invitrogen) of three DCLs are shown in S2 Table. The gene silencing effects were optimized through direct transfection of 8 μM siRNA. Briefly, primordia were first treated with synthetic siRNAs and incubated at 25 °C for 24 h. The transfected primordia were then treated with the same concentration of siRNAs and incubated for another 24 h. After the double transfection, total RNA samples of the control groups, untreated primordium and unrelated transfection (primordium with RNase-free water injection), and DCL knockdown strains were harvested. The remaining gene expressions of knockdown strains compared with the control groups were measured by quantitative real-time PCR using primers listed in S1 Table. Primers were designed to detect sequences between the sites of siRNA directed cleavage or at the target site of siRNA.

### MilRNA target prediction and functional annotation

Most miRNAs bind to the 3’-UTR of their target mRNAs to down-regulated their gene expressions. Nevertheless, there are no general rules for the complementarity between fungal milRNAs and no 3’-UTR data have been determined for *C*. *cinerea* [22]. A database was constructed from the 1,000 bp downstream sequences of the stop codon of all genes in the *C*. *cinerea* genome for miRNA target prediction, as in the milRNA studies on other fungi (*N*. *crassa*, *T*. *ressei* and *P*. *marneffei*) [15, 17, 18, 22]. As no prediction algorithms have yet been developed for fungi, three separate tools (PITA, miRanda and microTar) were used here to predict the potential targets of validated milRNAs in order to minimize false positive results [57–60]. These three tools focus primarily on the thermodynamic considerations—miRanda predicts the stability of miRNA/target duplexes based on the hybridization energy of the binding site in 3’-UTR, whereas PITA and microTar take both the hybridization energy and accessibility of the 3’-UTR into account. Although the ranking criteria of miRanda is slightly different from the other two algorithms, PITA and miRanda has been demonstrated previously to give comparable results[61]. Therefore, the overlapped targets predicted by these three tools were chosen as the most likely putative targets.

Additional filtering steps were applied to select for putative targets with annotated biological functions using Gene Ontology (GO) terms, Eukaryotic Orthologous Groups (KOG) groups, KEGG orthologs (KO), and KEGG biological pathways [62, 63, 64]. The GO terms of targets were assigned using BLAST2GO (version 2.4.2) with default parameters. The KOG groups were assigned by RPS-BLAST (E-value cut-off of 1.00E-3). The KO and KEGG biological pathways were assigned with the KEGG Automatic Annotation Server (KAAS) using all available fungal species as the representative gene set and the bidirectional best hits method (BBH). GO enrichment analysis was done with topGO (Release 3.7) using one-sided Fisher’s exact test [65]. KOG enrichment analysis was done with the HYPGEOM.DIST function in Microsoft Excel using one-sided Fisher’s exact test. KEGG enrichment analysis was done with DAVID 6.8 (http://david.ncifcrf.gove/summary.jsp) [66, 67]. A p-value smaller than 0.05 was considered as significant. To further identify target mRNAs that likely interact with milRNA in vivo, annotated putative targets with similar expression patterns to the corresponding milRNAs between the two developmental stages were selected based on previously published microarray data of *C*. *cinerea* [29]. Functional targets with a fold change ≤ 0.5 and > 0.5 at MYC compared to PRI were considered as putative milRNA targets of cci-milR-12c and cci-milR-13e-5p, respectively.

## Results

### Identification of sRNAs in *C*. *cinerea* by high-throughput sequencing

**Fig 1:**
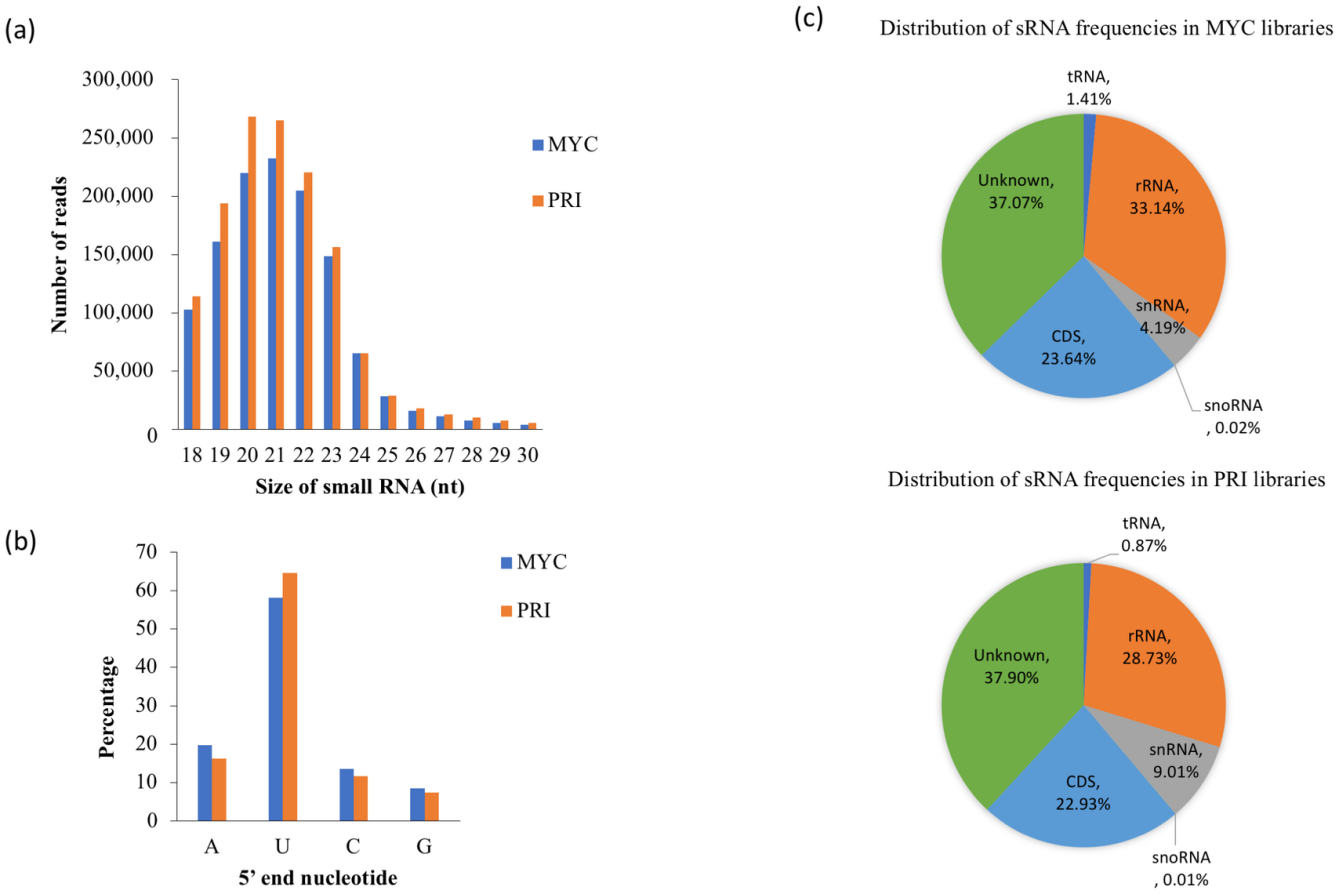
Features of sRNAs uncovered in *C*. *cinerea*. (a) Size distribution and (b) 5’ end nucleotide frequency of sRNAs in mycelial (MYC) and primordium (PRI) stages. (c) Pie charts showing the distribution of sRNA frequencies in two MYC and PRI libraries.

The general features of sRNA species identified in MYC and PRI of *C*. *cinerea* are shown in Table 1. A total of 16,925,614 and 17,490,760 raw reads were obtained from MYC and PRI, respectively. A total of 1,354,235 and 1,379,040 unique sRNA reads (18–30 nt) were obtained from the MYC and PRI stages, respectively. A total of 152,835 and 135,648 rRNAs, and 15,890 and 12,280 tRNAs were included in the unique clean reads of the MYC and PRI samples, respectively. The majority of sRNA reads in *C*. *cinerea* were derived from the coding regions, followed by rRNA and a tiny amount from tRNA and snoRNAs (Fig 1c). The percentage of rRNA-derived sRNAs of *C*. *cinerea* was similar to that of *S*. *sclerotiorum and T*. *ressei*, but differed from *F*. *oxysporum* and *N*. *crassa* in that about half of the sRNA reads were derived from rRNA [15, 16, 17, 20]. Most sRNA clean reads from both stages were 20–22 nt in length (Fig 1a) and displayed a strong preference for 5’ uracil (Fig 1b).

**Table 1:** General features of sRNA sequencing of *C*. *cinerea* in two developmental stages.

	MYC	PRI
Total reads	16,925,614	17,490,760
Trimmed reads^a^	9,543,074	11,505,899
Filtered reads^b^	9,188,318	10,924,076
Unique reads	2,837,886	3,696,516
Mapped reads^c^	2,685,051	3,574,137
rRNA reads	152,835	135,648
tRNA reads	15,890	12,280
sRNA reads (18–30 nt)	1,354,235	1,379,040
Conserved miRNAs	0	0
Predicted milRNAs	22	20

^a^ Raw reads were filtered to remove low quality reads, adaptor and primer sequences.

^b^ Trimmed reads were filtered to remove short reads < 18nt.

^c^ Against the reference genome of *C*. *cinerea* (NCBI assembly accession: AACS00000000). MYC: mycelium library, PRI: primordium library.

### Prediction and identification of potential milRNAs in *C*. *cinerea*

**Fig 2:**
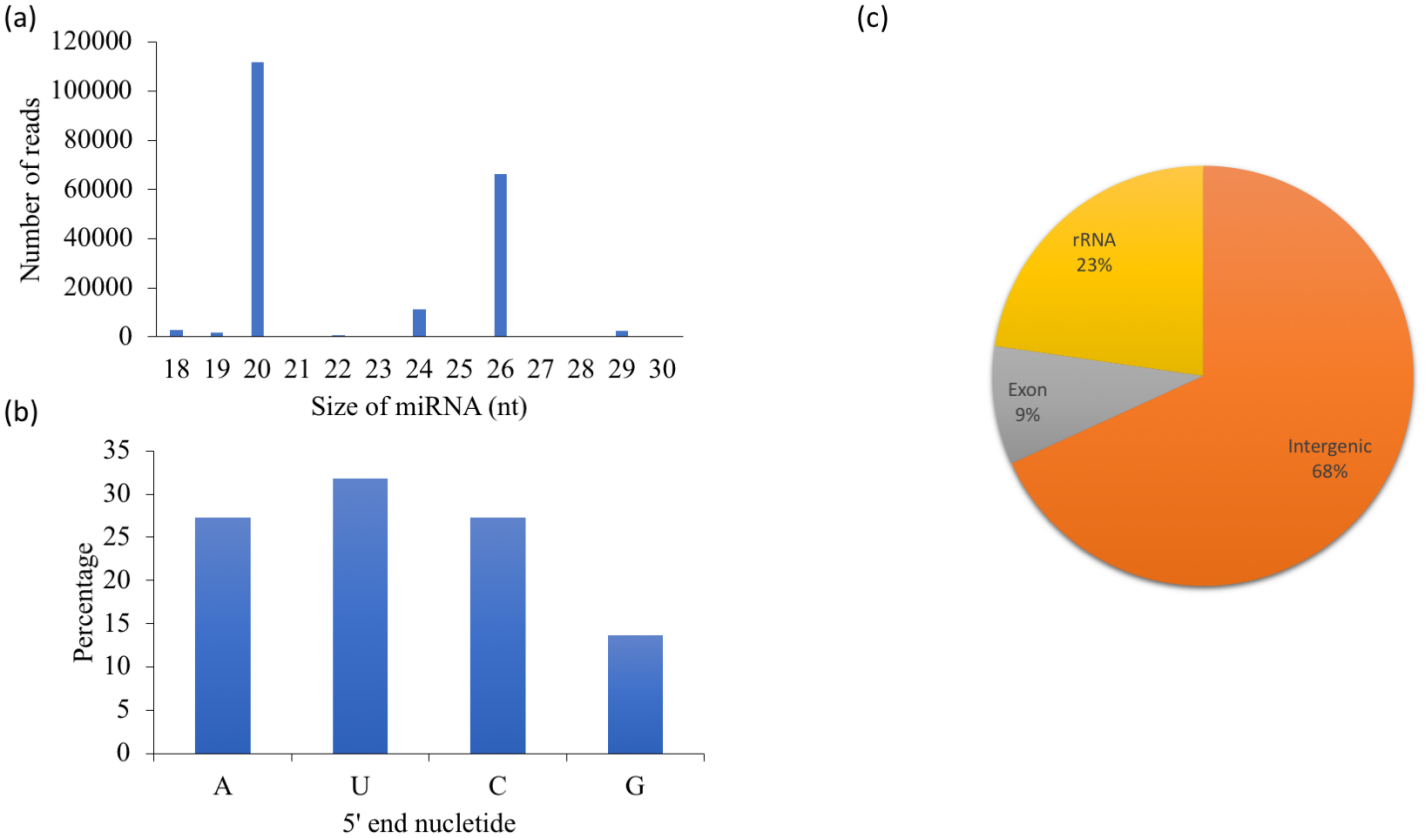
Characterization of putative milRNA candidates in *C*. *cinerea*. (a) Size distribution, (b) 5’ end nucleotide frequency and (c) annotation gene loci of the 22 putative milRNAs.

Twenty-two putative milRNA candidates were identified in *C*. *cinerea*. Most of them appeared in both MYC and PRI, but cci-milR-1 and cci-milR-2, which were only presented in MYC. The read counts and sequences of the milRNAs are listed in Table 2. The 20 and 26 nt classes were the most abundant groups in the milRNA candidates (Fig 2a). Guanine dominated the 5’ end nucleotide with weak superiority (Fig 2b). As with canonical miRNAs in animals and plants, most of the milRNAs in *C*. *cinerea* were derived from the intergenic region (68%), including five from rRNA (23%) and two from exon (9%) (Fig 2c). As for the gene locations, half of the putative milRNAs dwelled on the assembled chromosomes (Table 2). Six putative milRNAs predicted in *C*. *cinerea* were located within a short distance on the U413 contig, similar to milRNAs in animals and plants, which usually appear in clusters [68].

**Table 2:** Twenty-two predicted milRNAs in *C*. *cinerea*.

milRNA ID	Locus of mature milRNA^a^	Strand	milRNA sequence (5’ to 3’)	Length (nt)	Read number in MYC	Read number in PRI
cci-milR-1	Chr_1:2276359-2276380	+	CTGGATGGTGTGGGAGTTGCT	21	179	0
cci-milR-2	Chr_4:444870-444895	+	ACGAAGCAGTCGGCGCACTGGACGT	25	33	0
cci-milR-3	Chr_6:260570-260591	-	ATGAGCTCAGCGGTTATCCGAT	22	11	15
cci-milR-4a	Chr_7:1973680-1973700	+	TTTGCGGTGATGACTGACGT	20	1,152	3,469
cci-milR-4b	Chr_7: 2383250-2383270	+	TCAGTCATCACCGCAAACCA	20	1,143	4,096
cci-milR-5-3p	Chr_9:6467-6488	-	TTCTTAGGAATATCGGCCAGAC	22	3.5	3
cci-milR-5-5p	Chr_9:6494-6515	-	CTTGGCACTCGGTCGATATTCC	22	6.5	3
cci-milR-6	Chr_9: 2295986-2296004	-	CATCTGTCCTTCCCGCTGC	19	16	16
cci-milR-7	Chr_11:1942223-1942244	+	TCTTCCGAACCTCTTGATAGCT	22	25	25.5
cci-milR-8	Chr_12:1120052-1120072	-	CTGACTTCTGCCAGCCATTCT	21	33	29
cci-milR-9	Chr_12:1565625-1565643	+	TGCTTGGACTTCTATGGC	18	1,379	1,434
cci-milR-10	U377:434-457	-	GTGAAAAGACATAGAGGGTGTAGA	24	8,925	2,518
cci-milR-11	U382:2844-2863	-	GAAAAGTGACGGCTCATCCC	20	44	73.5
cci-milR-12a	U401:686-705	-	ATTGACACGGCTGGGCTTTT	20	14.5	16
cci-milR-12b	U401:1048-1069	+	TGAGTAGAATGGTCCCTGTCCC	22	220	232
cci-milR-12c	U401:4487-4512	-	GGCGCCGTTGAAATACCACTACCTTT	26	3,724	50,524
cci-milR-13a	U413:2349-2377	-	ATATTTGGTATTTGCGCCTGTCCGATCGG	29	2,321	248
cci-milR-13b	U413:2422-2441	+	ATAACACTCCATCAGTAGGG	20	2	2
cci-milR-13c	U413:2964-2989	-	TGTGAAAAGACATAGAGGGTGTAGAA	26	9,147	2,863
cci-milR-13d	U413:3111-3130	-	CTAATTAGTGACGCGCATGA	20	6,832	418
cci-milR-13e-3p	U413:3387-3405	-	ACCTCTAGATGGACCCCGC	19	900	823

^a^ Positions according to the reference genome of *C*. *cinerea* (NCBI assembly accession: AACS00000000). MYC: mycelium library, PRI: primordium library.

Although no conserved milRNA of animals and plants was found in *C*. *cinerea*, one homolog of cci-milR-12c was identified in another mushroom forming basidiomycete fungus, *Laccaria bicolor* (GSE9784). This homolog was absent in *Phanerochaete chrysosporium*, *Postia placenta*, two other *Pleurotus ostreatua*, and *Agaricus bisporus* [69–73]. Sequence of the cci-milR-12c precursor (pre-milR-12c) was BLAST searched against the *L*. *bicolor* EST database [69], and only sequences with no mismatches on the seed region of mature cci-milR-12c and with fewer than three mismatches to the downstream sequence of the seed region were regarded as homologs [74]. The absence of milRNA homologs of animals and plants in *C*. *cinerea* indicates evolutionary divergence of miRNA genes among these three kingdoms, coinciding with most of the fungal milRNAs [16, 17, 20].

### Validation and characterization of milRNA expression patterns

**Fig 3:**
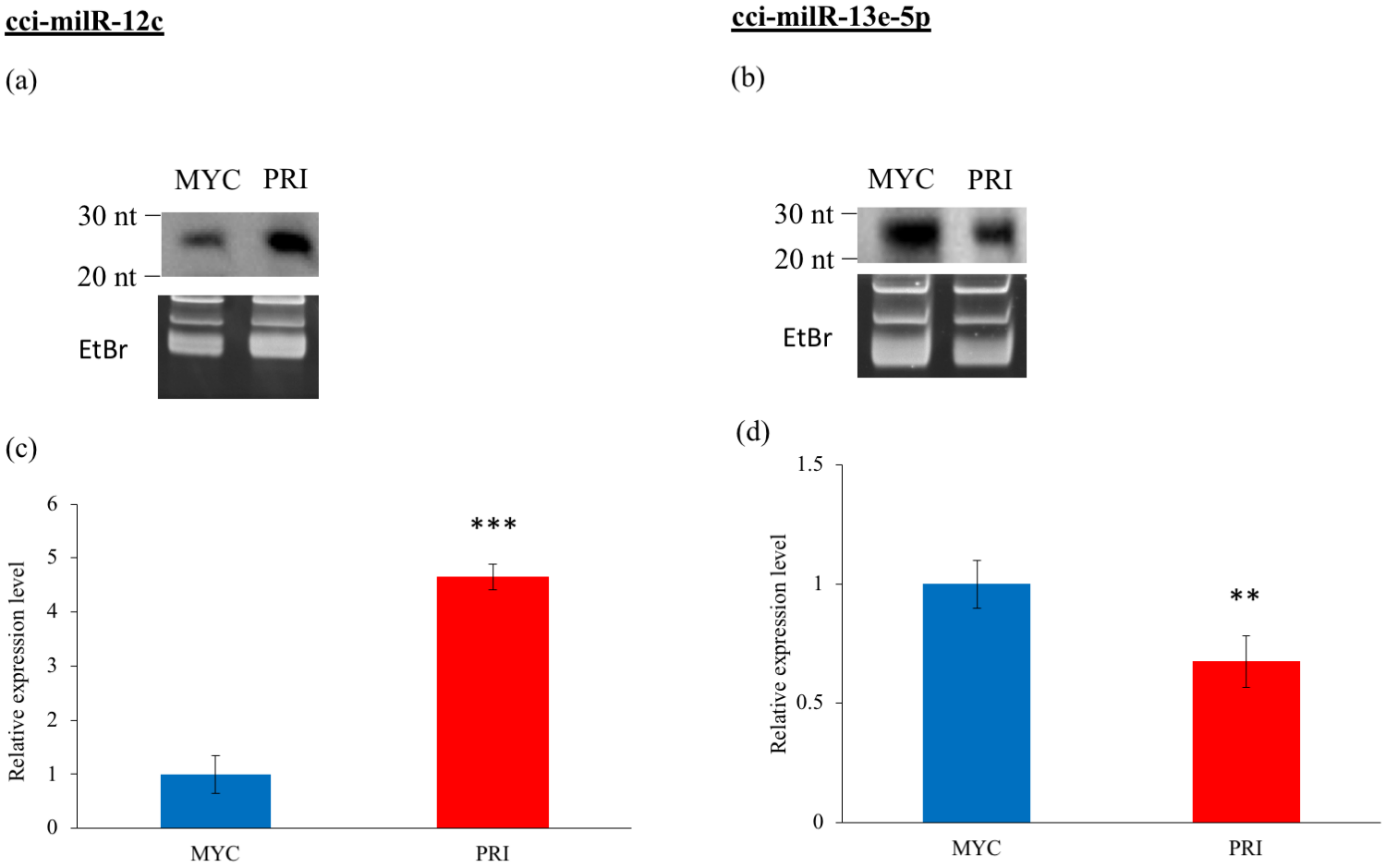
Validation of two milRNA candidates by northern blot and RT-qPCR. MYC: mycelium, PRI: primordium. Northern blot of sRNA samples showed the presence of (a) cci-milR-12c and (b) cci-milR-13e-5p in both developmental stages of *C*. *cinerea*. The top panel shows northern blots probed with the milRNA-specific DIG probes. The 15% denaturing gel stained with ethidium bromide (EtBr) in the bottom panel indicates equal loading of RNA samples. RT-qPCR results show the expression levels of (c) cci-milR-12c and (d) cci-milR-13e-5p in both developmental stages. Results were obtained from three independent experimental replicates and were significantly different between stages. ** p < 0.01, *** p< 0.001.

**Fig 4:**
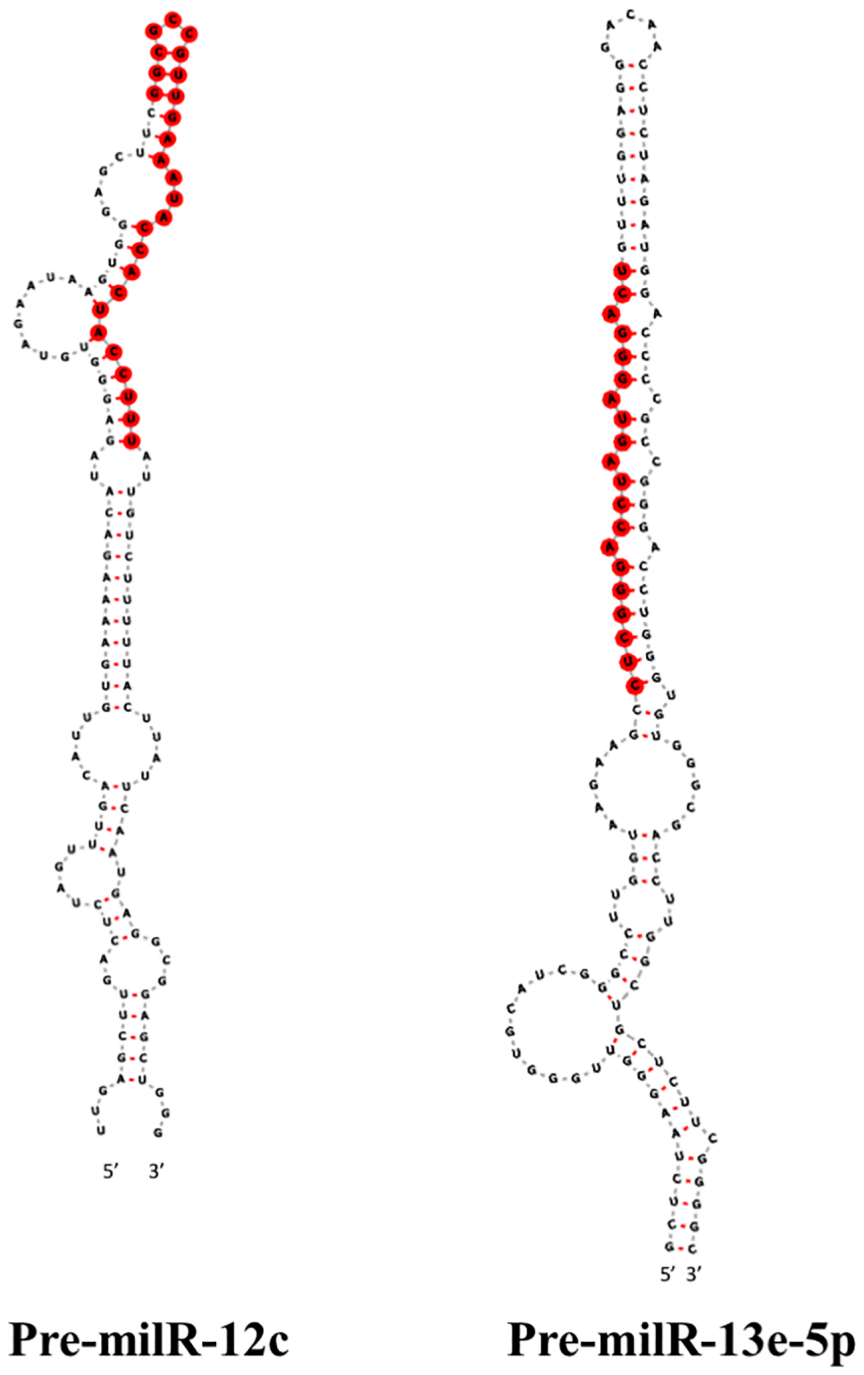
Predicted secondary structures of milRNA precursors. The predicted structures of pre-milR-12c and pre-milR-13e-5p with mature milRNA sequences are labeled in red.

Northern blot and RT-qPCR were used to validate the presence and to examine the expression levels of putative milRNAs in the two developmental stages. Two out of 22 milRNA candidates, cci-milR-12c and cci-milR-13e-5p, were verified using northern blot. Their expression patterns during this developmental transition are shown in Fig 3. By contrast, other putative milRNAs were not detected by Northern blot analyses carried out here. cci-milR-12c showed a higher expression in PRI (fold change >2), indicating that this milRNA candidate was differentially expressed in the early developmental stage. By contrast, the expression level of cci-milR-13e-5p in MYC was only slightly higher than that in PRI. The hairpin precursors of the two validated milRNAs are shown in Fig 4. Annotation of the gene loci of these two miRNAs indicated that the cci-milR-12c gene is located on an unassembled contig and cci-milR-13e-5p is derived from the intergenic region, based on the genome assembly data (AACS00000000) [22].

Although no annotated genes were identified in the locus of mature cci-milR-12c, there was a significant hit of pre-milR-12c to a eukaryotic rRNA sequence (accession: RF02543) when searching the precursor sequence on the Rfam database (E-value = 1.6e-26). Nucleotide sequence search also revealed that the pre-milR-12c sequence matched to the 28S rDNA locus of *C*. *cinerea* (E-value = 8e-62). Similar to rRNA-derived miRNAs found in human, the location of rRNA genes recovered here was distinct from that of the rRNA genes and cci-milR-12c might be generated during processing of the transcribed rRNA gene [75].

### Identification of DCL, AGO and QDE-2 proteins and characterization of their expression patterns in MYC and PRI

**Fig 5:**
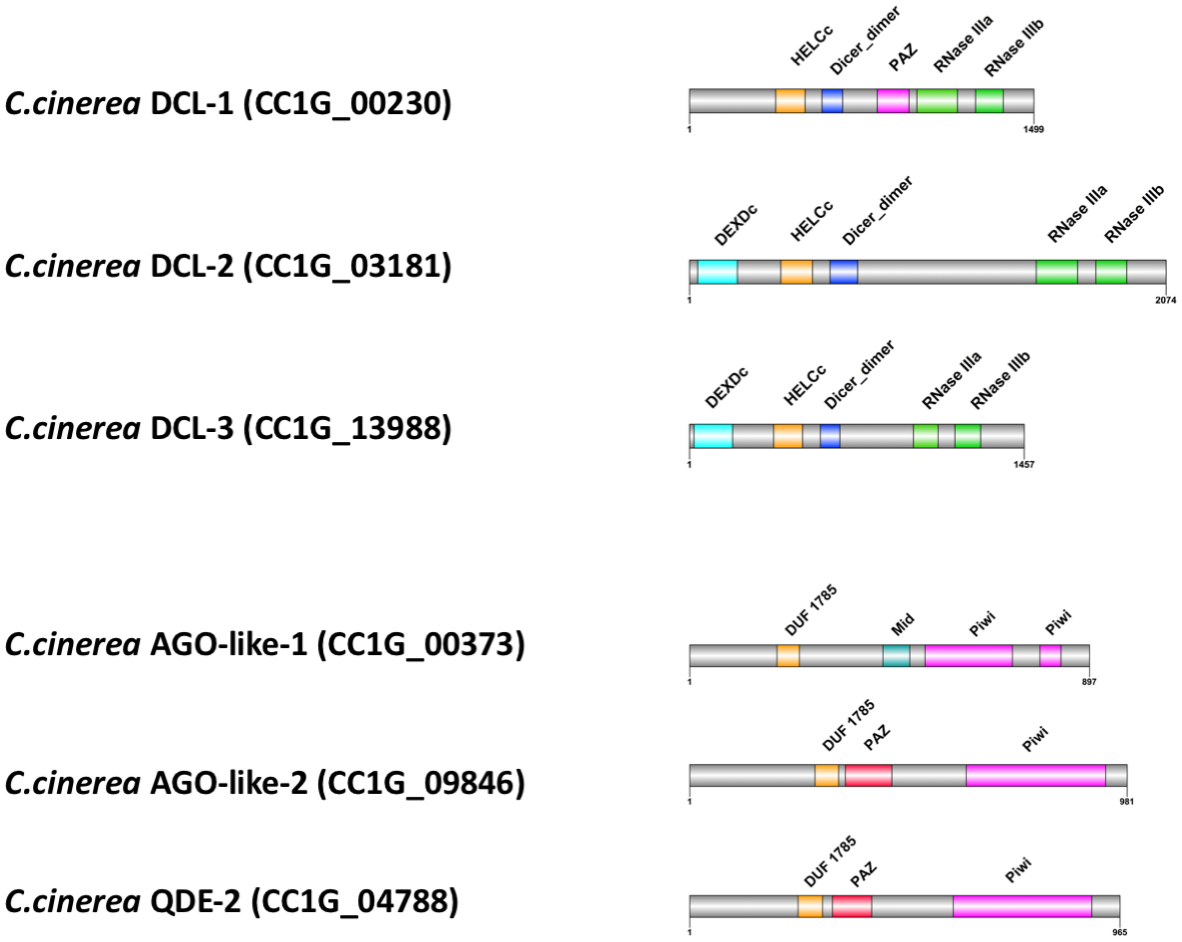
Schematic 2D domain architecture of Dicer and AGO proteins in *C*. *cinerea*. The grey bars represent the full protein sequences and the colored boxes represent identified functional domains.

Dicer and AGO are effector proteins known to participate in miRNA biogenesis in animals and plants [76]. QDE-2 protein is an AGO shown to be involved in the pre-miRNA cleavage in *N*. *crassa* and its homologs have also been found in various fungal species [15, 18, 19]. Based on homolog search of the *N*. *crassa* DCL proteins against the *C*. *cinerea genome*, three DCL proteins were found (Fig 5) [15, 22]. Furthermore, an AGO (CC1G_00373), an AGO-like (CC1G_09846) and a QDE-2 (CC1G_04788) proteins have been annotated in *C*. *cinerea* [22]. The AGO and AGO-like genes CC1G_00373 (3,979 bp in length) and CC1G_09846 (3,457 bp in length) encode mRNAs for protein of 897 and 981 amino acids, respectively. The QDE-2 gene (CC1G_04788) is 3,438 bp in length encoding mRNA for protein of 965 amino acids. All AGO family proteins predicted in *C*. *cinerea* have at least one of the two characteristic domains of AGO protein: PAZ and Piwi domain. The mRNAs of Dicer protein homologs (CC1G_00230, CC1G_03181, CC1G_13988) encode 1499, 2074 and 1457 amino acid residues, respectively. Interestingly, only one of the *C*. *cinerea* DCLs (CC1G_00230) contained the PAZ domain, which is present only in mushroom-specific DCLs but not in other fungal DCLs. Although PAZ is a conserved domain in both Dicer and many AGO family proteins, it cannot be found in the annotated AGO proteins (CC1G_00373) in *C*. *cinerea* (Fig 5) [77, 78]. In general, the domain organization of DCL, AGO, AGO-like and QDE-2 proteins of *C*. *cinerea* was similar to that of *N*. *crassa*, except that there is no PAZ domain in the AGO protein (S4 Table).

**Fig 6:**
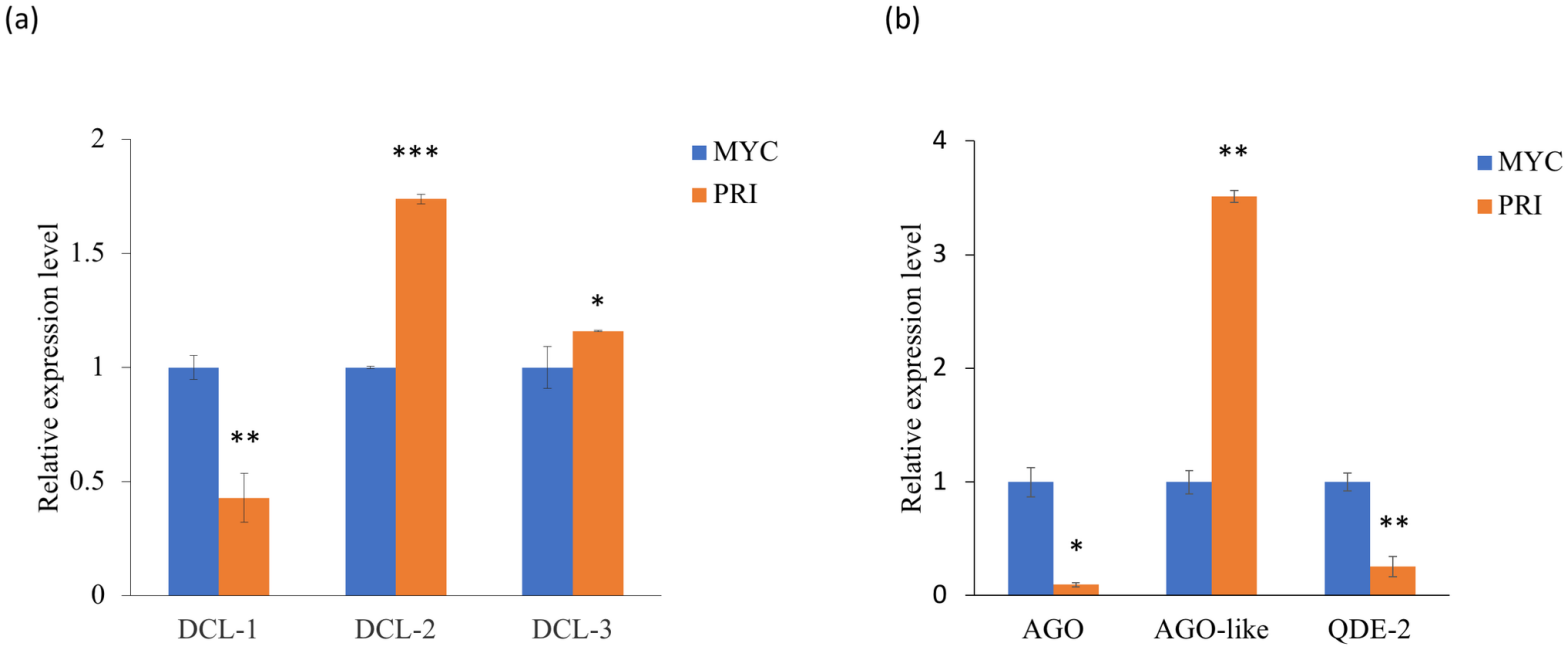
Relative mRNA expression levels of DCL and AGO proteins. The expression levels of (a) Dicer-like (DCLs), (b) AGO, AGO-like and QDE-2 proteins in mycelium (MYC) and primordium (PRI) stages. Results were obtained from three independent experimental replicates and were significantly different between stages. *p < 0.05, ** p < 0.01, *** p< 0.001.

RT-qPCR was used to examine the mRNA expression levels of the protein homologs. The results are shown in Fig 6. DCL-2 and DCL-3 showed higher expression levels in PRI, however, but DCL-1 was down regulated in PRI (Fig 6a). For the AGO homologs, the expression levels of AGO and QDE-2 proteins were significantly lower in PRI (Fig 6b). Similar to the expression levels of cci-milR-12c, DCL-2 and DCL-3, AGO-like proteins were expressed significantly higher in PRI than in MYC. Therefore, DCL-2 or DCL-3 and AGO-like proteins are more likely involved in the biogenesis of cci-milR-12c. On the contrary, AGO or QDE-2 and DCL-1 are more likely related to the higher expression of cci-milR-13e-5p in MYC.

### Phylogenetic analysis of DCL and AGO homologs

**Fig 7:**
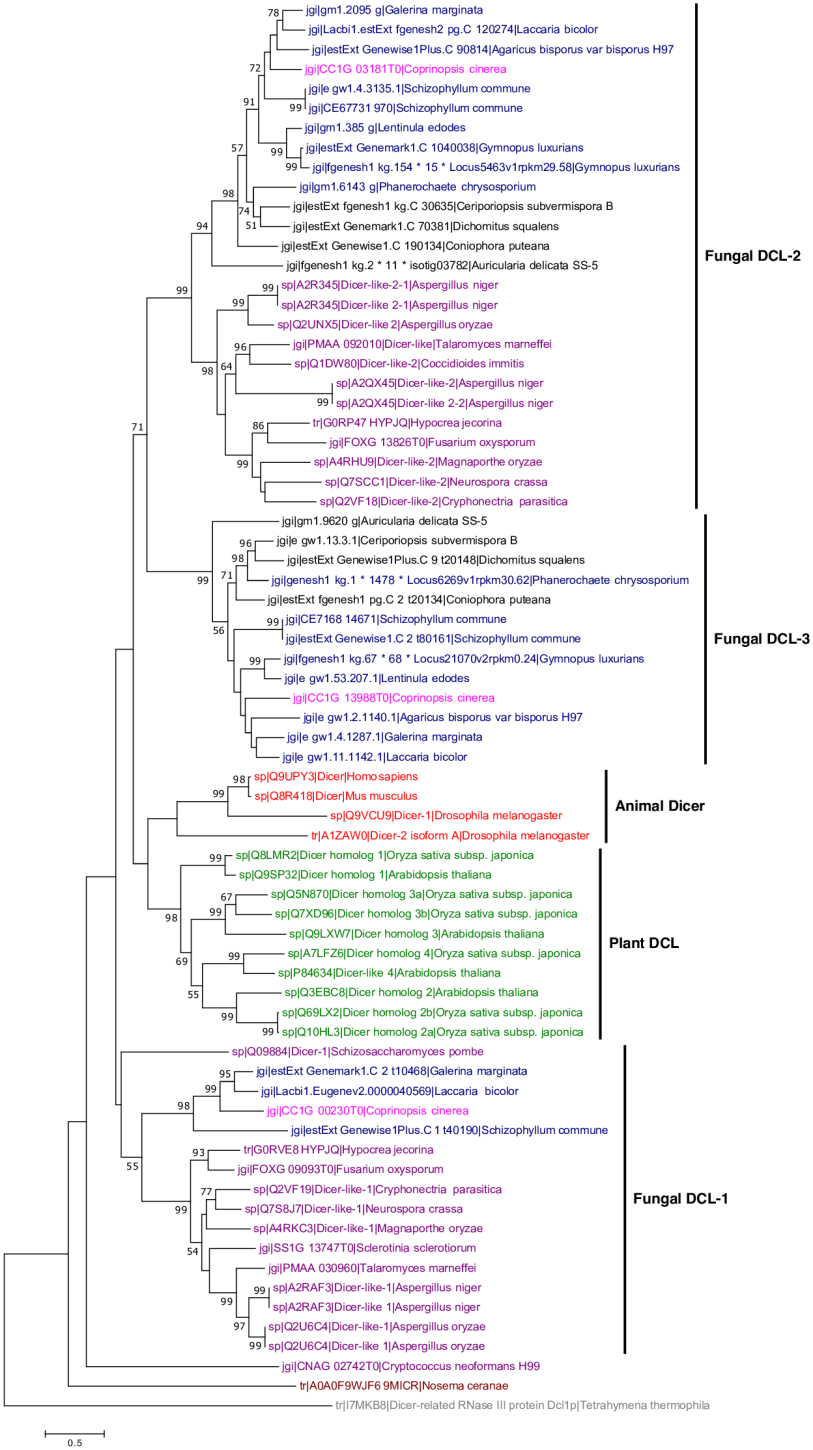
Phylogenetic tree of DCL proteins in animals, plants and fungi. The tree was constructed using the maximum likelihood method. Different groups (with colors) are animals (red), plants (green), non-mushroom forming basidiomycetes (black), mushroom-forming basidiomycetes (blue), *Coprinopsis cinerea* (pink), ascomycetes (purple), unicellular fungi (brown), protozoan (grey). The protozoan *Tetrahymena thermophile* was used as an outgroup. Bootstrap values were calculated from 1000 replicates and only values ≥50% are shown here. The scale bar represents 0.5 substitutions per nucleotide position.

**Fig 8:**
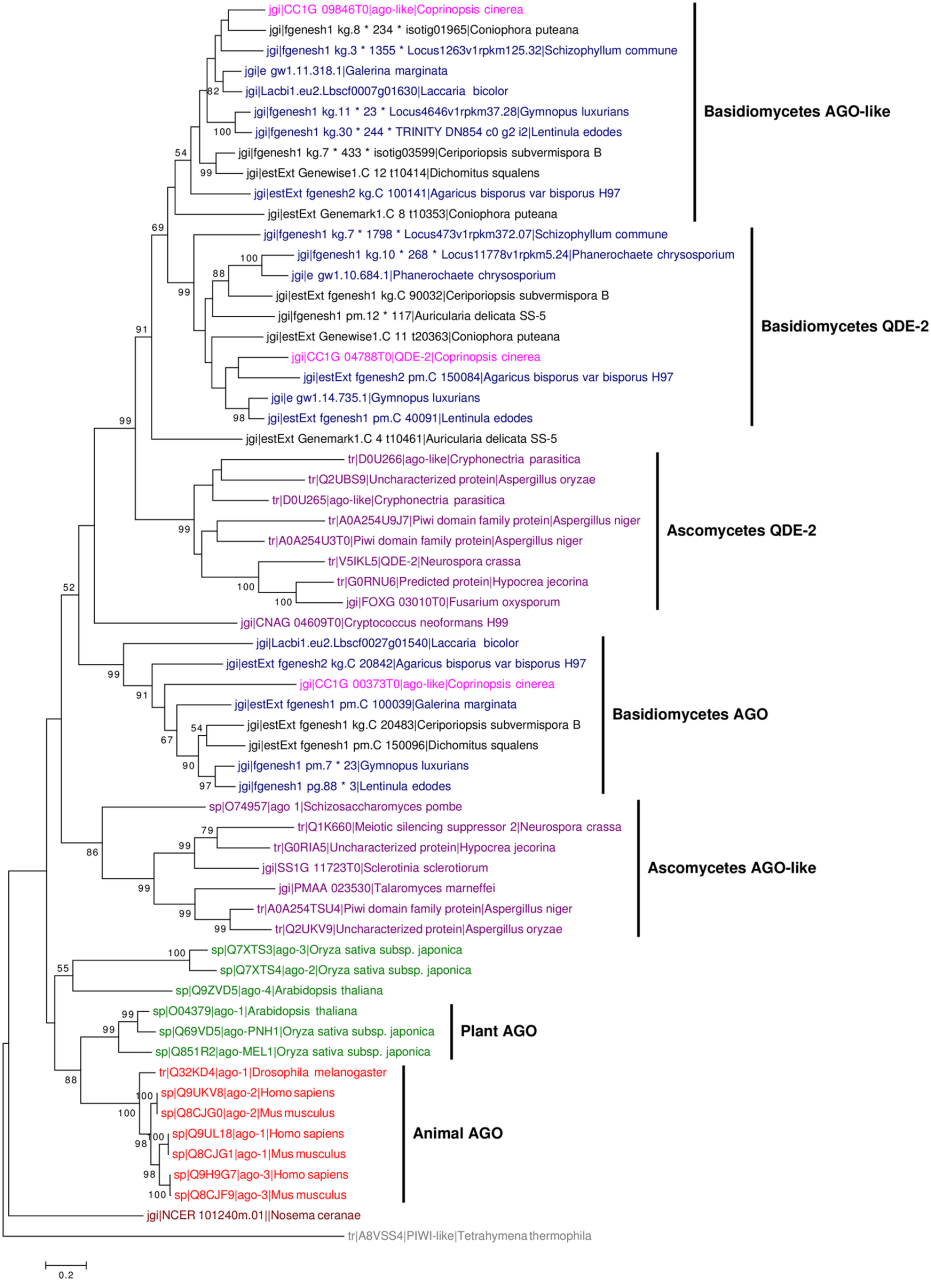
Phylogenetic tree of AGO proteins in animals, plants and fungi. The tree was constructed using the maximum likelihood method. Different groups (with colors) are animals (red), plants (green), non-mushroom forming basidiomycetes (black), mushroom-forming basidiomycetes (blue), *Coprinopsis cinerea* (pink), ascomycetes (purple), unicellular fungi (brown), protozoan (grey). The protozoan *Tetrahymena thermophile* was used as an outgroup. Bootstrap values were calculated from 1000 replicates and only values ≥50% are shown here. The scale bar represents 0.2 substitutions per nucleotide position.

Phylogenetic analysis of DCL and AGO proteins showed that both proteins duplicated early in the eukaryotic lineage and evolved independently in animals, plants and fungi (Figs 7 and 8). DCL and AGO homologs in *C*. *cinerea* were closely related to those in other basidiomycetes. Most of the mushroom forming fungi possess three DCLs, while other fungi contain only two DCLs. Besides, one DCL (CC1G_00230) of *C*. *cinerea* was grouped with DCLs of other mushroom forming basidiomycetes, namely *Galerina marginata*, *Laccaria bicolor* and *Schizophyllum commune*, and this group of mushroom-specific DCL protein has not been discussed previously (Fig 7) [79]. These results suggest that Dicer proteins duplicated and diversified early in the eukaryotic lineage.

Based on the phylogenetic analysis results and protein domain architecture of the annotated DCL-1 and DCL-2 proteins among ascomycete and basidiomycete fungi, the three predicted DCL homologs in *C*. *cinerea* were named in this study [79]. A novel group of DCL homolog in basidiomycete in the phylogenetic tree was revealed which was further supported by the protein domain comparison among annotated fungal DCLs. Since all of the annotated DCL-1 proteins of fungi possess the type III restriction enzyme domain instead of the DEAD/DEAH box helicase domain (as in DCL-2 proteins), and as the PAZ domain was only found in mushroom-forming basidiomycetes (*L*. *bicolor* and *G*. *marginata*), the putative DCL genes CC1G-13988, CC1G_03181 and CC1G_00230 were named as DCL-1, DCL-2 and DCL-3, respectively (S4 Table).

### The expression levels of milRNAs in the DCL knockdown strains

Sequence-specific siRNA duplexes were used to knockdown individual DCL mRNA. The efficiency of knockdown of DCL mRNA following double transfection and the expression patterns of the two validated milRNAs in DCL knockdown strains are summarized in S1 Fig. Primordium transfected with siRNAs showed 60–80% knockdown of DCL mRNA transcript abundance compared to the two control groups. A DIG-labelled probe specific for cci-milR-12c detected three bands in the control, with approximate sizes of 25/26, 40 and 50 nt on northern blot. The cci-milR-13e-5p-specific probe also revealed three bands on the blot, with sizes of about 20, 30, 40 nt. The ~20 nt bands were similar in size to the predicted cci-milR-12c and cci-milR-13e-5p, suggesting that they are the mature milRNAs. By constrast, the intermediate RNAs ~30–50 nt in size are likely the precursors of milRNAs (pre-milRNAs). However, the signals of the two mature milRNAs of DCL knockdown strains were similar to those of the controls. Given that the expression of DCL mRNAs was not completely abolished using knockdown, the roles of DCLs in milRNA biogenesis cannot be confirmed here.

### Prediction and functional annotation of milRNA targets

**Fig 9:**
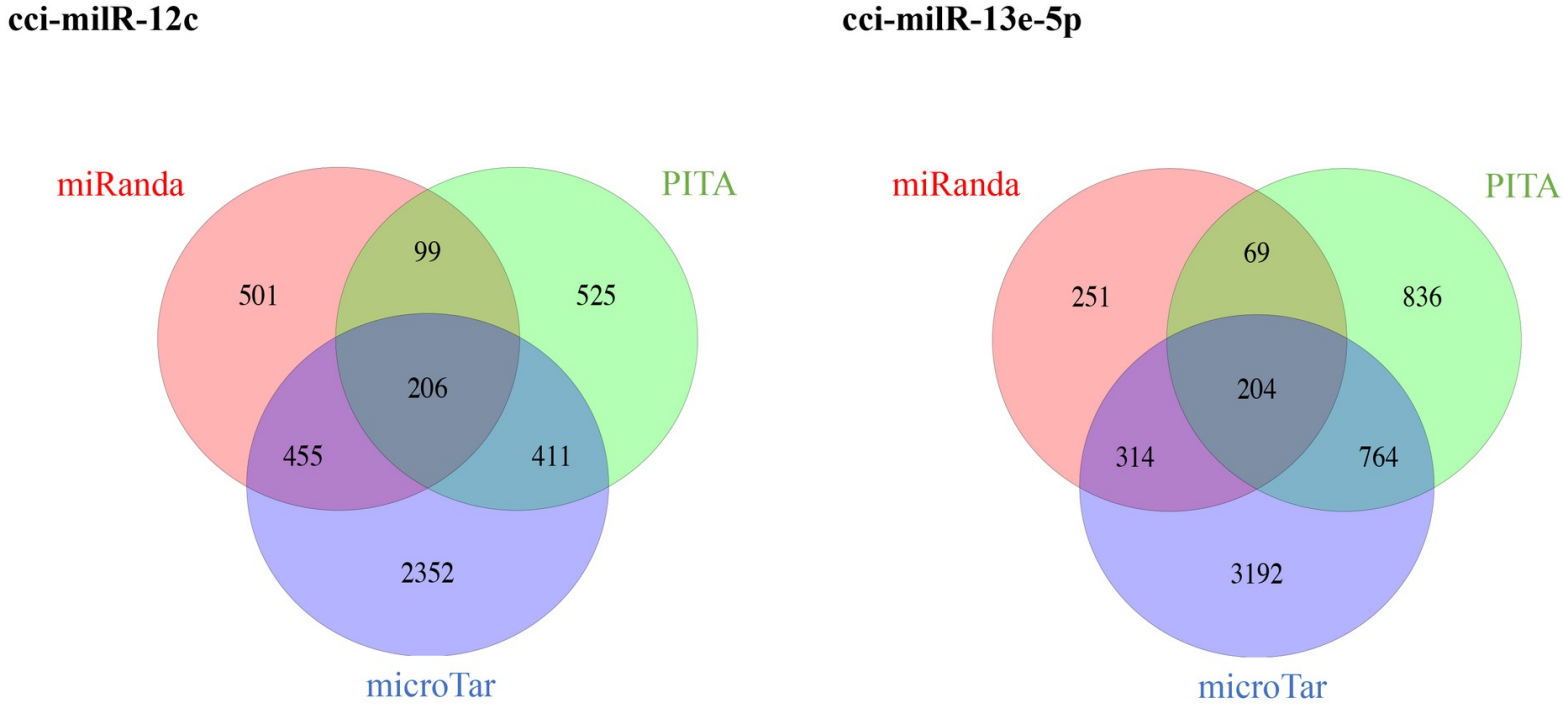
Venn diagram showing the distribution of the number of putative milRNA targets predicted by miRanda, PITA and microTar.

Computational prediction of milRNA targets was carried out based on three different algorithms: miRanda, PITA and microTar, to minimize false-positive results. Each prediction algorithm predicted a few hundreds to thousands of target genes for each milRNA. A larger number of targets was predicted by microTar due to the fact that miRanda and PITA rely on evolutionary conservation to select functional targets whereas microTar discerns milRNA targets by calculating the duplex energies without taking into account the conservation of miRNA targets [56–59]. The number of overlapped targets is shown in a Venn diagram (Fig 9). There were 206 and 204 common targets of cci-milR-12c and cci-milR-13e-5p, respectively. Of these, 143 and 140 were annotated with functional GO, KOG terms or fruiting body related genes (data not shown). Given that the expression patterns of milRNA are similar to their targets and two milRNAs showed higher expression in MYC and PRI respectively, the expression levels of putative targets during the transition from MYC to PRI were used for the last filtering step. As a result, 15 and 133 functional genes were selected as the putative targets of cci-milR-12c and cci-milR-13e-5p, respectively (S3 Table).

**Fig 10:**
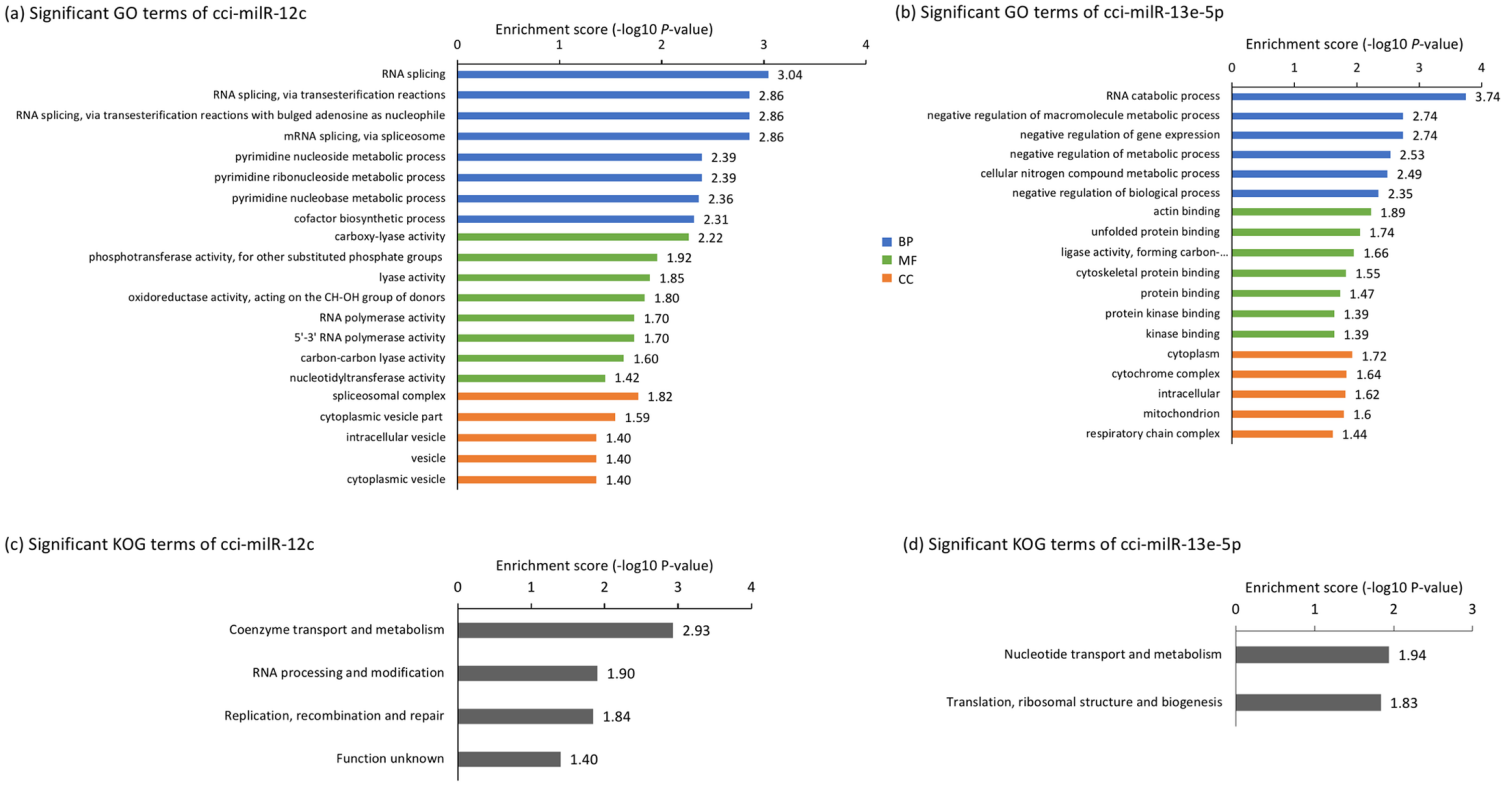
GO and KOG enrichment analyses of two validated milRNAs. GO enrichment analysis of the predicted targets of (a) cci-milR-12c and (b) cci-milR-13e-5p. GO terms were classified with Blast2GO into three major categories and representative results of GO enrichment with topGO using Fisher’s exact test, p-value < 0.05. BP: Biological process, MF: Molecular function, CC: Cellular component. KOG enrichment analysis of the predicted targets of (c) cci-milR-12c and (d) cci-milR-13e-5p.

To fully understand the functions of the putative targets of milRNA, all the overlapped targets of each milRNA were annotated using GO terms, KOG terms and KEGG pathway. Both GO and KOG enrichment analyses showed that the targets of cci-milR-12c were enriched in “RNA processing and metabolism”, especially RNA splicing. About 60% of enriched GO terms were in this category (Fig 10a and 10c). On the contrary, the targets of cci-milR-13e-5p were enriched in “nucleotide transport and metabolism” including RNA catabolic processes, and “translation, ribosomal structure and biogenesis” (Fig 10b and 10d).

Additional functional annotation of putative milRNA targets was performed by searching the eukaryotic homologs in the KOG database (Fig 10c and 10d). Putative targets of cci-milR-12c were assigned to 22 groups and cci-milR-13e-5p were assigned to 23 groups. The category “RNA processing and modification” was the largest group annotated to cci-milR-12c targets, followed by “Replication, combination and repair” and “Coenzyme transport and metabolism”. “Nucleotide transport and metabolism” and “Translation, ribosomal structure and biogenesis” were the enriched KOG terms annotated to the targets of cci-milR-13e-5p.

Overall, results from the GO and KOG term annotations of the two validated milRNAs were similar. Since none of the annotated KEGG pathways for milRNA targets were found to be significantly enriched, when using p-value cut-off at 0.05. Therefore, the results of KEGG analysis were not included here.

Interestingly, some enriched GO terms of the milRNA targets of cci-milR-13e-5p were closely related to the developmental processes in fungi, including ligase activity, actin, unfolded protein, and protein kinase binding. The expression levels of target genes under these categories passed the fold change threshold in the last filtering step of target prediction (S3 Table). Although there were a limited number of putative targets of cci-milR-12c that were annotated with the functional GO and KOG terms and were differentially expressed in MYC, some fruiting body related genes were included, including fungal pheromone, hydrophobin and cytochrome P450. Taken together, these results suggest that milRNAs may play an important role on regulating different metabolic pathways and facilitating the cellular developments during the early developmental transition in *C*. *cinerea*.

## Discussion

In this study, we constructed sRNA libraries and identified milRNAs of *C*. *cinerea* at two different developmental stages. Characteristics of *C*. *cinerea* milRNA populations similar to those in animals and plants and the presence of core proteins of miRNA biogenesis in *C*. *cinerea* suggest that milRNAs in mushrooms may be produced in similar pathways to those in animals and plants. The functional analysis of milRNA targets also demonstrates the potential regulatory roles of milRNAs in fruiting body development (S2 Fig).

The expression patterns of milRNAs hint at their biological functions across different biological processes. Here, we identified putative targets that exhibited a negative correlation in expression profiles with milRNAs during the transition from MYC to PRI, some of which were related to fruiting body formation. cci-milR-12c potentially controls the vegetative growth of hyphae by targeting the fungal pheromone, stage-specific hydrophobin and nucleotide metabolic process. Fungal pheromone is responsible for initiating septal dissolution and clamp-cell fusion during the transition from monokaryotic to dikaryotic state, whereas hydrophobin regulates morphogenesis in fungi, particularly in fruiting body development of basidiomycetes [26, 80, 81]. It has also been reported that different sets of hydrophobins are employed by mushroom forming basidiomycetes in different developmental stages [81, 82]. By contrast, most of the putative targets of cci-milR-13e-5p were related to macromolecule metabolism and protein binding. For instance, cci-milR-13e-5p targeted protein kinase that has been predicted to respond to nutrient depletion in fruiting body initiation, especially FunK1, which can only be found in multicellular fungi. In addition, the up-regulated heat shock proteins in PRI are response to the lower temperature for fruiting body development (25°C) than that of mycelial cultivation (37 °C) [22, 23, 83–86]. Therefore, cci-milR-13e-5p is more likely to regulate the dynamic structural changes, carbon, protein and nucleotide metabolism, in response to an increased demand of DNA synthesis, protein synthesis and turnover from the mycelial to primordium stage [28]. Results suggest that milRNAs may play a role in controlling the drastic transcriptomic and morphological changes during fruiting body initiation.

Phylogenetic analysis results of DCLs, AGO, AGO-like and QDE-2 proteins and the fact that no miRNA homologs of animals and plants were identified in the *C*. *cinerea* genome support the claim that miRNAs may evolve independently among animals, plants and fungi [15, 35, 87, 88, 89]. Our results also indicate an early duplication and diversification of Dicer proteins followed by a lineage-specific loss of PAZ domain in fungi. In evolutionary terms, DCL-3 (CC1G_00230) is evolutionary closely related to other mushroom forming fungi and is the only DCL in *C*. *cinerea* that contains the PAZ domain, which has only been found in mushroom forming basidiomycetes, such as *G*. *marginata* and *L*. *bicolor* [69, 90]. The PAZ domain recognizes the 3’ 2-nt overhang of pre-miRNA during miRNA biogenesis and the specific distance between the anchoring site of PAZ and RNase III domain is used to determine the milRNA product size [91]. However, this functional domain is absent in other fungal species included in the phylogenetic analysis, suggesting that different molecular mechanisms are adopted by DCLs without the PAZ domain to produce milRNAs with heterogeneity in length. Indeed, size heterogeneity of fungal milRNAs has been reported in *N*. *crassa* and *F*. *oxysporum* [15, 16]. Furthermore, homologs of cci-milR-12c were found in another mushroom, *L*. *bicolor*. Since the PAZ domain-containing DCL was only identified in mushroom-forming fungi, further studies should investigate its uniqueness in mushrooms and if the milRNA produced by this homolog function differently to those from the other homologs.

Given that miRNAs are generally produced from a hairpin precursor by Dicer, the accumulation of pre-miRNAs can be detected in organisms with impaired Dicer function [13]. Change of miRNA expression patterns is an indicator of the participation of Dicer in its biogenesis. Although homologous recombination has been found in *C*. *cinerea*, gene knockouts are difficult to achieve due to the high efficiency of non-homologous DNA end joining [92, 93]. Therefore, an alternative gene silencing method, dsRNA-mediated gene knockdown, which was successfully used in the study of *C*. *cinerea* strains #326 *(A43mut B43mut pab1-1)*, was used in this study [93]. However, cci-milR-12c and cci-milR-13e-5p were still produced—corresponding RNA bands of these milRNAs were detected in northern blot, with a ~70% knockdown efficiency of DCLs. It is possible that milRNAs are efficiently produced, even when the expression levels of the DCLs are extremely low. Future experimental work is needed to investigate the roles of PAZ-containing DCLs in milRNA biogenesis of mushrooms and to determine if the milRNA homologs play a regulatory role in other mushroom forming fungi.

## Conclusions

Our findings have demonstrated differential post-transcriptional regulatory roles of milRNAs in different developmental stages of the mushroom forming fungus *C*. *cinerea* and identified the milRNA potential targets involved in fruiting body formation, providing new insights into the regulatory mechanisms of fruiting body development and the potential functions of milRNAs in fungi. Moreover, we have found putative core miRNA biogenesis proteins, Dicer and AGO, in the *C*. *cinerea* genome. Phylogenetic analysis showed that these proteins were more closely related to those in other fungal species than to those in animals and plants. However, the roles of DCLs, AGO and QDE-2 proteins in the biogenesis of *C*. *cinerea* milRNAs cannot be shown here. Altogether, these results serve as the foundation for further evolutionary developmental studies of fungi and contribute to the phylogenetic occurrence of miRNA-mediated regulatory system among different kingdoms.

## Supporting information

S1 Fig Effect of DCL knockdown on miRNA expression. (a) RT-qPCR expression levels of DCLs obtained in DCL knockdown strains after normalization against the untreated primordium (control). Results were obtained from three independent experimental replicates. The treatment samples were significantly different from the control samples. *p < 0.05, ** p < 0.01. Northern blot of sRNA samples shows the presence of (b) cci-milR-12c, (c) cci-milR-13e-5p and their precursors in all the knockdown strains. The top panels show the northern blots probed with milRNA-specific DIG probes. The 15% denaturing gels stained with ethidium bromide (EtBr) in the bottom panels indicate equal loading of RNA samples.
(TIF)

S2 Fig Schematic summary of the milRNA study in *C*. *cinerea*. (TIF)

S1 Table Primers used in RT-qPCR for expression determination of core biogenesis proteins. F: forward primer, R: reverse primer.
(PDF)

S2 Table Stealth siRNA duplexes used in DCLs knockdown assays. S: sense strand, AS: antisense strand of siRNA duplexes.
(PDF)

S3 Table Target prediction of two validated milRNAs in *C*. *cinerea*. Predicted targets of (a) cci-milR-12c and (b) cci-milR-13e-5p. Norm_MYC and Norm_PRI represent normalized expression levels at the mycelium (MYC) and primordium (PRI) stages based on previously published microarray data of *C*. *cinerea* [25]. Targets were predicted by using miRanda, PITA and microTar and selected by several rounds of functional annotation. Description and domain information are downloaded from http://www.broadinstitute.org.
(PDF)

S4 Table Domain information about the annotated Dicer-like proteins in different ascomycetes and some predicted Dicer-like homologs in *C*. *cinerea*, *L*. *bicolor* and *G*. *marginata*. ^1^ Accession/ Protein IDs were extracted from the database of Uniprot or JGI. ^2^ ResIII: Type III restriction enzyme, res subunit. Helicase_C: Helicase conserved C-terminal domain. Dicer-dimer: Dicer dimerization domain. Ribonuclease_#: Ribonuclease III domain. DEAD: DEAD/DEAH box helicase.
(PDF)
