# Supplementary figures and images for: Discovery of microRNA-like RNAs during early fruiting body development in the model mushroom Coprinopsis cinerea
Source: PLoS One. 2018 Sep 19;13(9):e0198234. doi: 10.1371/journal.pone.0198234 (PMC6145500; doi:10.1371/journal.pone.0198234)

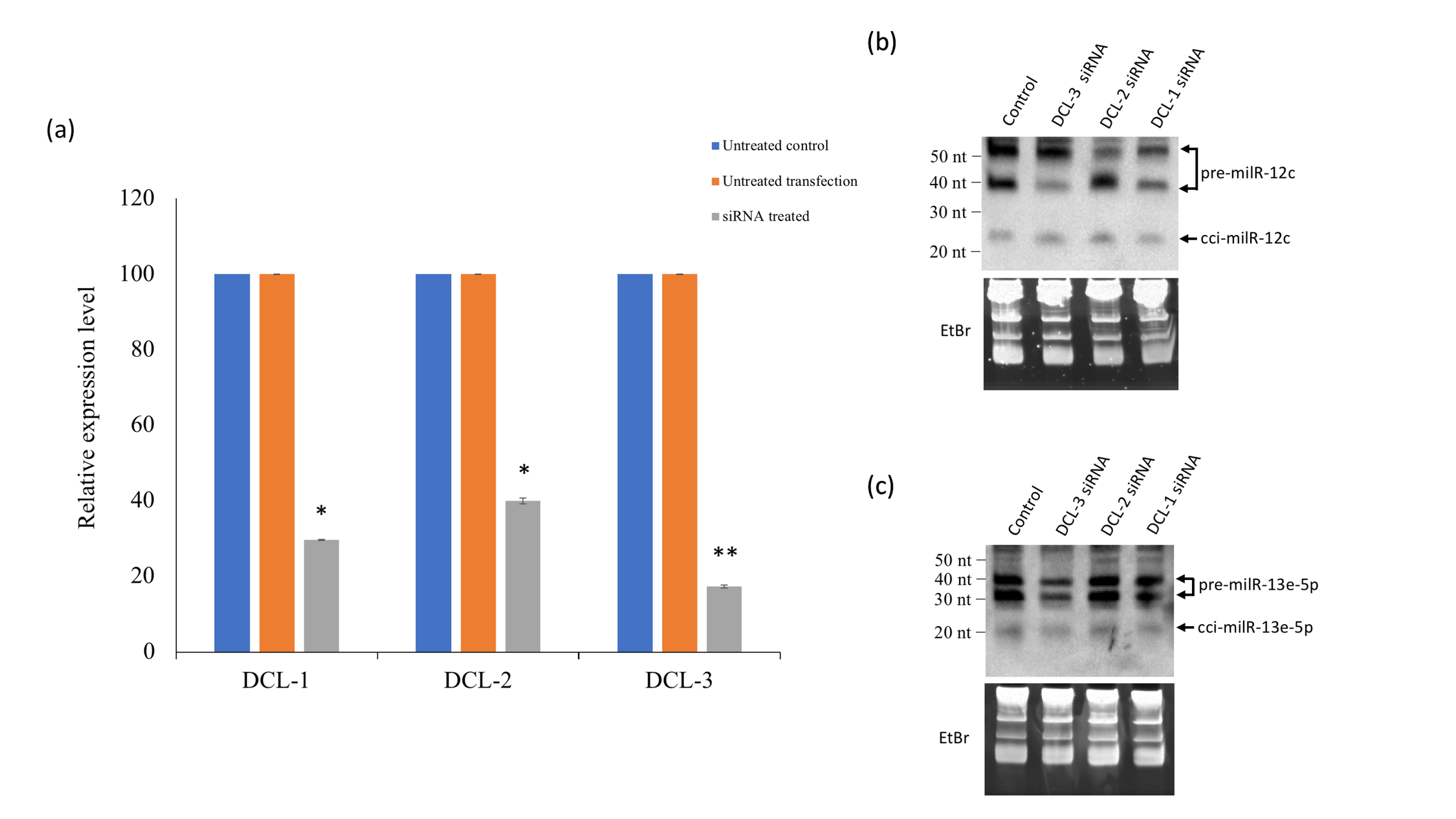

Supplement: S1 Fig — (a) RT-qPCR expression levels of DCLs obtained in DCL knockdown strains after normalization against the untreated primordium (control). Results were obtained from three independent experimental replicates. The treatment samples were significantly different from the control samples. *p < 0.05, ** p < 0.01. Northern blot of sRNA samples shows the presence of (b) cci-milR-12c, (c) cci-milR-13e-5p and their precursors in all the knockdown strains. The top panels show the northern blots probed with milRNA-specific DIG probes. The 15% denaturing gels stained with ethidium bromide (EtBr) in the bottom panels indicate equal loading of RNA samples. (TIF) [file pone.0198234.s001.tif]

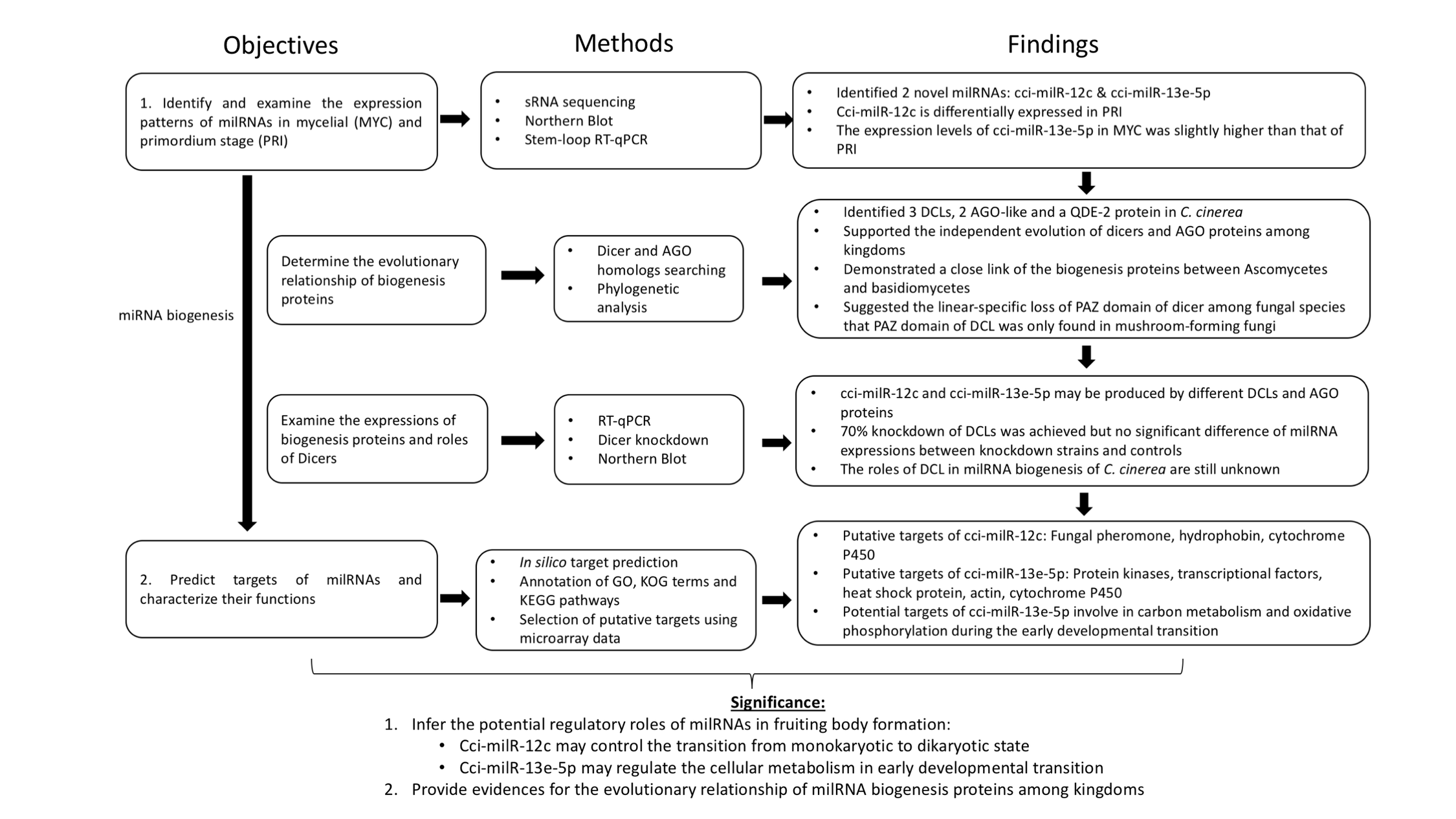

Supplement: S2 Fig — (TIF) [file pone.0198234.s002.tif]
