# Supplementary material for: Discovery of microRNA-like RNAs during early fruiting body development in the model mushroom Coprinopsis cinerea
Source: PLoS One. 2018 Sep 19;13(9):e0198234. doi: 10.1371/journal.pone.0198234 (PMC6145500; doi:10.1371/journal.pone.0198234)
Supplement: S1 Table — F: forward primer, R: reverse primer. (PDF) [file pone.0198234.s003.pdf]

Gene ID	Description	Primer sequences (5' to 3')	Amplicon size (bp)
CC1G_00230	Dicer-like-1	F: GAGAACCGAATGCCAGGACA<br>R: CGACAGGGCAGAAGTTGTCT	115
CC1G_03181	Dicer-like-2	F: CTCGAGACGCTCGGTGATAC<br>R: CCACAGCGTGATCCTTCCTT	112
CC1G_13988	Dicer-like-3	F: ATCGTCACGAAGGCCAACTT<br>R: GCAACGGATGCAATCTCGAC	121
CC1G_00373	AGO-like-1	F: CGCTCTCCGATTTCTCTCCC<br>R: ATCTCTGTCGCCCTACCCTT	101
CC1G_09846	AGO-like-2	F: CGCTCTCCGATTTCTCTCCC<br>R: ATCTCTGTCGCCCTACCCTT	126
CC1G_04788	QDE-2	F: TTCGTCAGGAAAGGTCTGGC<br>R: GCTTGATCCCCGGGTAGATG	115
