# Supplementary material for: Discovery of microRNA-like RNAs during early fruiting body development in the model mushroom Coprinopsis cinerea
Source: PLoS One. 2018 Sep 19;13(9):e0198234. doi: 10.1371/journal.pone.0198234 (PMC6145500; doi:10.1371/journal.pone.0198234)
Supplement: S2 Table — S: sense strand, AS: antisense strand of siRNA duplexes. (PDF) [file pone.0198234.s004.pdf]

Description	siRNA sequences (5' to 3')
DCL-1-siRNA-1	S: CCGUGGCGAAUAAUCUCCAUCCGAA<br>AS: UUCGGAUGGAGAUUAUUCGCCACGG
DCL-1-siRNA-2	S: CGGCAUAUAUCUGUUCGAAGCCUUU<br>AS: AAAGGCUUCGAACAGAUAUAUGCCG
DCL-1-siRNA-3	S: CCCAAAGGCUCUGUCUGAUGUGAUA<br>AS: UAUCACAUCAGACAGAGCCUUUGGG
DCL-2-siRNA-1	S: UAUACGAUAUCGAUCCGCCUGAUUA<br>AS :UAAUCAGGCGGAUCGAUAUCGUAUA
DCL-2-siRNA-2	S: CCUAUGUGGCAUGAGGGCUAUUUGA<br>AS: UCAAAUAGCCCUCAUGCCACAUAGG
DCL-3-siRNA-1	S: CCGUUUCGAAUAAGUUUCUCCUUCA<br>AS: UGAAGGAGAAACUUAUUCGAAACGG
DCL-3-siRNA-2	S: CACCCUCGCUUACCUGGCAAUCAAA<br>AS: UUUGAUUGCCAGGUAAGCGAGGGUG
