# Supplementary material for: Discovery of microRNA-like RNAs during early fruiting body development in the model mushroom Coprinopsis cinerea
Source: PLoS One. 2018 Sep 19;13(9):e0198234. doi: 10.1371/journal.pone.0198234 (PMC6145500; doi:10.1371/journal.pone.0198234)
Supplement: S3 Table — Predicted targets of (a) cci-milR-12c and (b) cci-milR-13e-5p. Norm_MYC and Norm_PRI represent normalized expression levels at the mycelium (MYC) and primordium (PRI) stages based on previously published microarray data of C. cinerea [25]. Targets were predicted by using miRanda, PITA and microTar and selected by several rounds of functional annotation. Description and domain information are downloaded from http://www.broadinstitute.org. (PDF) [file pone.0198234.s005.pdf]

(a)

Gene ID	Norm_MYC	Norm_PRI	Description	Domain
CC1G_08185	7479.8	583.6	fungal mating-type pheromone	Pheromone;
CC1G_04037	9592.7	986.1	cytochrome P450	p450;
CC1G_10206	7108.3	792.5	predicted protein	Hydrophobin;
CC1G_14509	2056.2	229.5	fungal mating-type domain-containing protein-containing protein	Pheromone;
CC1G_08424	2089.4	411.8	predicted protein	-
CC1G_03745	2728.8	636.8	dihydroorotate oxidase	-
CC1G_07014	4706.7	1164.8	hypothetical protein	-
CC1G_14935	3040.5	843.2	hypothetical protein	-
CC1G_05404	8917.8	2714.8	hypothetical protein	-
CC1G_08106	9252.3	3725.8	predicted protein	-
CC1G_08106	9252.3	3725.8	predicted protein	-
CC1G_14922	1180.2	515.5	predicted protein	-
CC1G_05923	9388.4	4193.2	LAP1	Peptidase_M28;PA;
CC1G_06049	7538.8	3510.8	ankyrin repeat domain-containing protein 44	Ank;
CC1G_09790	913.7	428.3	aryl-alcohol oxidase	GMC_oxred_N;GMC_oxred_C;

(b)

Gene ID	Norm_MYC	Norm_PRI	Description	Domain
CC1G_03982	6474.5	3502.5	predicted protein	-
CC1G_02554	8448.2	4683.5	Utr2p	Glyco_hydro_16;
CC1G_02242	6636.7	3743.8	other/FunK1 protein kinase	-
CC1G_05295	2585.1	1479.7	alpha-L-arabinofuranosidase A	Alpha-L-AF_C;
CC1G_06799	5750.2	3456.4	hypothetical protein	-

CC1G_07035	158.7	96.2	other/FunK1 protein kinase	-
CC1G_02289	4786.8	2934.3	hypothetical protein	-
CC1G_03113	2431.3	1569.4	short chain dehydrogenase	adh_short;
CC1G_01902	8105.5	5426.8	predicted protein	-
CC1G_12456	10597.4	7401.2	hypothetical protein	Deme6;
CC1G_01261	9143.4	6549.2	AGC/RSK protein kinase	Pkinase;
CC1G_11447	8098.3	6041.5	glycosyl hydrolase family 16	-
CC1G_09642	13434.8	10029	hypothetical protein	MFS_1;
CC1G_00850	10696.6	8030.9	DnaJ domain-containing protein	DnaJ;
CC1G_05183	7589.9	5720.1	AGC/PKA protein kinase	Pkinase;
CC1G_02630	16913.3	13342.6	sugar transporter	SP;Sugar_tr;MFS_1;
CC1G_00125	7118.5	5726.5	dUTP pyrophosphatase	dUTPase;dut;
CC1G_03275	4038.3	3250.1	predicted protein	DnaJ;
CC1G_09360	2736.5	2230.6	Cip2	-
CC1G_04658	2237	1846.5	predicted protein	F-box;
CC1G_01400	5612.4	4689.5	tRNA (guanine-N(1)-)-methyltransferase	tRNA_m1G_MT;
CC1G_09907	3994.5	3342.2	peptide chain release factor 1	prfA;RF-1;PCRF;
CC1G_03837	12248.3	10459.1	ubiquitin-protein ligase	WW;HECT;C2;
CC1G_06894	16374.1	14033.5	heat shock protein	HSP70;prok_dnaK;
CC1G_09824	12020.6	10363.9	predicted protein	-
CC1G_00694	13650	11907.3	mitochondrial ribosomal protein subunit L2	Ribosomal_L2;Ribosomal_L2_C;rplB_bact;
CC1G_13846	6447.9	5636.5	hypothetical protein	AAA_5;
CC1G_03087	12175.7	10809.6	metalloreductase	NAD_binding_1;Ferric_reduct;
CC1G_10651	5037.8	4473.2	hypothetical protein	-
CC1G_11217	5229.1	4658.3	predicted protein	-

CC1G_00726	5542.8	4947.4	NGG1 interacting factor 3	NIF3;TIGR00486;
CC1G_01041	3783.5	3415.5	predicted protein	-
CC1G_03447	12217.4	11057.2	C-3 sterol dehydrogenase	NAD_binding_4;3Beta_HSD;Epimerase;
CC1G_08881	9149.3	8306.4	co-chaperone	DnaJ;
CC1G_11465	10660.4	9738.8	isocitrate dehydrogenase	nadp_idh_euk;Iso_dh;
CC1G_00077	6078.8	5554.2	hypothetical protein	UCR_UQCRX_QCR9;
CC1G_08232	17092.9	15677.6	actin	Actin;
CC1G_12346	5221.5	4801.2	hypothetical protein	Acetyltransf_1;
CC1G_03705	12983.1	11982.5	6-phosphogluconate dehydrogenase	gnd;NAD_binding_2;6PGD;
CC1G_03449	17817.8	16526.1	fatty acid desaturase	FA_desaturase;
CC1G_08304	12178.9	11297.5	hypothetical protein	COX4;
CC1G_06506	10232	9543.3	Arg-6 protein	DUF619;Semialdhyde_dh;argC;AA_kinase;argB;
CC1G_03670	13352.2	12460.4	cyclin	Cyclin_C;Cyclin_N;
CC1G_05488	2466.8	2373.2	orotate phosphoribosyltransferase	pyrE;Pribosyltran;
CC1G_09782	8866.3	8548.6	cytochrome P450	p450;
CC1G_05544	2596.4	2509.6	nudix-type domain-containing protein 1 isoform p22	NUDIX;
CC1G_14619	5135.8	4980.9	YwlC protein	FMC1;TIGR00057;Sua5_yciO_yrdC;SUA5;
CC1G_10110	15584.8	15127.9	ubiquitin-carboxy extension protein fusion	Ribosomal_L40e;ubiquitin;
CC1G_09090	110	107.1	predicted protein	zf-MYND;
CC1G_11306	12701.7	12399.9	fatty acid elongase	ELO;
CC1G_04850	2127.9	2089.6	folylpolyglutamate synthetase and dihydrofolate synthetase	folC;
CC1G_01173	17238.5	17162.1	GTP-binding nuclear protein RAN	Miro;small_GTP;Ras;
CC1G_04130	87.2	87.1	alpha-ketoglutarate-dependent taurine dioxygenase	TauD;
CC1G_13742	16687	16693.8	calnexin	Calreticulin;

CC1G_01782	6282.2	6299.2	bifunctional purine biosynthetic protein ADE1	purM;purD;GARS_C;GARS_N;AIRS_C;GARS_A;AIRS;
CC1G_07633	6787.6	6895.3	predicted protein	NDUF_B7;
CC1G_01067	8167.4	8329.6	ATP dependent helicase	UPF1_Zn_bind;
CC1G_11064	14360.4	14653.8	nascent polypeptide-associated complex subunit beta	NAC;
CC1G_14862	6263.6	6407.3	trans-2-enoyl-CoA reductase	ADH_N;ADH_zinc_N;
CC1G_00505	7792.3	7993.5	RCC1 domain-containing protein	WD40;
CC1G_02540	12596	12923.7	cystathionine gamma-synthase	-
CC1G_12228	5857.1	6021.7	hypothetical protein	DUF2183;
CC1G_10540	12238.4	12667.3	ribosomal protein L4/L1	Ribosomal_S9;
CC1G_05320	8699.2	9017.8	g2/mitotic-specific cyclin cdc13	Cyclin_C;Cyclin_N;
CC1G_00835	6229	6586.7	MVP1 domain-containing protein	PX;
CC1G_07692	10797.9	11426.4	predicted protein	-
CC1G_07048	7115.7	7547.2	transcription factor iws1	IWS1_C;
CC1G_01755	7916.2	8407.3	hypothetical protein	Fungal_trans;Zn_clus;
CC1G_14239	9179.7	9883.8	hypothetical protein	DUF1709;PH;
CC1G_14637	5128.1	5589.7	predicted protein	-
CC1G_06434	5836.9	6369.4	DNA helicase	Helicase_C;rad25;ResIII;
CC1G_02898	7511.5	8230.6	eukaryotic translation initiation factor 3	WD40;
CC1G_10270	14790.1	16232.7	septin AspB	Septin;
CC1G_00705	8011	8812.1	coatomer beta subunit	Adaptin_N;Coatamer_beta_C;HEAT;
CC1G_08828	583.6	644.4	protein kinase subdomain-containing protein PKL/ccin4	-
CC1G_03661	2180.1	2458.6	predicted protein	-
CC1G_12022	8932.4	10167.2	vacuolar protein sorting 55 superfamily protein	Vps55;
CC1G_14620	6718.6	7662.2	tartrate transporter	MFS_1;

CC1G_03807	7443.5	8582.6	hypothetical protein	-
CC1G_06749	7367.7	8680.3	aspartate-tRNA ligase	aspS_arch;tRNA-synt_2;tRNA_anti;
CC1G_00663	5235.2	6195.5	dynactin	CAP_GLY;
CC1G_02916	7634.6	9153.4	predicted protein	zf-C3HC4;
CC1G_15293	6384.5	7660.5	eukaryotic translation initiation factor SUI1 family protein	unchar_dom_2;SUI1;
CC1G_04309	5617.7	6922.7	predicted protein	DUF292;
CC1G_06038	3608.2	4462.5	predicted protein	-
CC1G_06951	7504.5	9335.7	hypothetical protein	-
CC1G_11423	6017.3	7492.7	T-complex protein 1 subunit gamma	chap_CCT_gamma;Cpn60_TCP1;
CC1G_02516	3742.4	4665.2	mandelate racemase/muconate lactonizing enzyme	MR_MLE;
CC1G_14703	3956.9	4966.5	glutamyl-tRNA(Gln) amidotransferase	GatB_N;GatB_Yqey;gatB;GatB;
CC1G_07151	3928.7	4965.8	hypothetical protein	-
CC1G_06779	8838.2	11223.8	cytoplasmic protein	KH_1;
CC1G_00918	3880.2	4933.4	predicted protein	WD40;
CC1G_08033	5570.8	7086.6	flap endonuclease-1	XPG_N;XPG_I;
CC1G_06124	3024.4	3920.8	hypothetical protein	SH3_1;RhoGEF;PH;SH3_2;WH2;
CC1G_01803	3273.6	4251.4	replication factor C subunit 4	Rep_fac_C;AAA;
CC1G_06636	3556.9	4665.3	hypothetical protein	-
CC1G_12768	6561.8	8682	transcription factor	SNase;TUDOR;
CC1G_03758	4183.7	5588.5	homoserine kinase	thrB;GHMP_kinases_C;GHMP_kinases_N;
CC1G_00802	4475.8	6014.9	hypothetical protein	MFS_1;
CC1G_09040	613.6	827.7	predicted protein	Ank;
CC1G_09525	1697.7	2305.6	hypothetical protein	CBM_1;
CC1G_08031	2024.4	2750.4	mRNA decapping enzyme	DcpS;
CC1G_11297	4685.9	6401.5	ribose-phosphate pyrophosphokinase 3	Pribosyltran;ribP_PPkin;

CC1G_05719	8598.6	12350.8	alanine-glyoxylate transaminase	-
CC1G_12108	3091.9	4512.2	hypothetical protein	-
CC1G_07736	3390.6	4955.6	RAD52 DNA repair protein RADC	rad52;Rad52_Rad22;
CC1G_14672	2047.1	3006.4	GTP cyclohydrolase I	folE;GTP_cyclohydroI;
CC1G_00428	2937.1	4403.6	hypothetical protein	-
CC1G_13725	1247.3	1904.9	predicted protein	-
CC1G_04586	6031.1	9327.9	hypothetical protein	-
CC1G_01045	3279.3	5213.9	hypothetical protein	-
CC1G_04307	2985.7	4876	hypothetical protein	Sas10_Utp3;
CC1G_04051	1694	2834.7	glycosyl hydrolase family 16	-
CC1G_10564	3726.8	6558	predicted protein	-
CC1G_03337	3379.8	6064.8	cytoplasmic protein	salvage_mtnB;Aldolase_II;
CC1G_10341	2183.4	3924.7	hypothetical protein	-
CC1G_03968	3161.9	5856.4	RING finger protein	IBR;zf-CCCH;
CC1G_14702	1223.1	2274.5	oxidoreductase	-
CC1G_07166	299.9	578.7	hypothetical protein	CBM_1;
CC1G_06878	1989.3	3920.6	hypothetical protein	Nop16;
CC1G_01212	2467.6	5253.2	RNA-3'-phosphate cyclase	RTC_insert;18S_RNA_Rcl1p;RTC;
CC1G_03335	4567.9	11758.5	predicted protein	PAS_3;sensory_box;
CC1G_02750	1063	3127.9	predicted protein	zf-MYND;
CC1G_02255	3219.2	11853.2	hypothetical protein	-
CC1G_07510	222.3	829.1	predicted protein	-
CC1G_15570	936.4	3767.6	predicted protein	-
CC1G_03946	97.2	496.7	laccase 2	Cu-oxidase;Cu-oxidase_3;Cu-oxidase_2;
CC1G_14785	2801.5	15351.4	predicted protein	-

CC1G_13587	1743.8	11515.9	calcium binding protein Caleosin	Caleosin;
CC1G_01349	1165	8985.8	predicted protein	-
CC1G_01086	622.6	8740.4	hypothetical protein	Zn_clus;
CC1G_10721	626	10069.4	hypothetical protein	-
CC1G_09161	640.3	10507.3	hypothetical protein	MFS_1;
