# Supplementary material for: Discovery of microRNA-like RNAs during early fruiting body development in the model mushroom Coprinopsis cinerea
Source: PLoS One. 2018 Sep 19;13(9):e0198234. doi: 10.1371/journal.pone.0198234 (PMC6145500; doi:10.1371/journal.pone.0198234)
Supplement: S4 Table — 1 Accession/ Protein IDs were extracted from the database of Uniprot or JGI. 2 ResIII: Type III restriction enzyme, res subunit. Helicase_C: Helicase conserved C-terminal domain. Dicer-dimer: Dicer dimerization domain. Ribonuclease_#: Ribonuclease III domain. DEAD: DEAD/DEAH box helicase. (PDF) [file pone.0198234.s006.pdf]

Phylum	Species	Protein Name	Accession/Protein ID[1]	Pfam name[2]	Pfam id	Start position	End position	E-value
Ascomycota	*Neurospora crassa*	DCL1_NEUCR	Q7S8J7	ResIII	PF04851	124	292	1.90E-14
				Helicase_C	PF00271	461	577	4.80E-17
				Dicer_dimer	PF03368	654	740	1.60E-20
				Ribonuclease_3	PF00636	1099	1206	5.70E-20
				Ribonuclease_3	PF00636	1295	1423	3.80E-19
		DCL2_NEUCR	Q7SCC1	DEAD	PF00270	108	200	1.10E-07
				Helicase_C	PF00271	404	526	1.40E-16
				Dicer_dimer	PF03368	597	695	2.60E-19
				Ribonuclease_3	PF00636	998	1112	1.90E-17
				Ribonuclease_3	PF00636	1196	1359	1.80E-12
	Magnaporthe oryzae	DCL1_MAG07	A4RKC3	ResIII	PF04851	111	280	4.10E-16
				Helicase_C	PF00271	452	563	2.80E-18
				Dicer_dimer	PF03368	639	724	2.20E-17
				Ribonuclease_3	PF00636	1083	1189	4.30E-17
				Ribonuclease_3	PF00636	1280	1406	3.00E-17
		DCL2_MAGO7	A4RHU9	DEAD	PF00270	67	201	9.90E-17
				Helicase_C	PF00271	475	539	8.90E-17
				Dicer_dimer	PF03368	609	708	1.40E-19
				Ribonuclease_3	PF00636	1024	1127	1.60E-17
				Ribonuclease_3	PF00636	1205	1358	1.20E-12
	Coccidioides immitis	DCL1_COCIM	Q1DKI1	ResIII	PF04851	101	269	2.60E-13
				Helicase_C	PF00271	434	547	6.40E-16
				Dicer_dimer	PF03368	623	709	1.40E-20
				Ribonuclease_3	PF00636	1224	1338	1.90E-22
		DCL2_COCIM	Q1DW80	DEAD	PF00270	93	260	6.00E-18
				Helicase_C	PF00271	440	564	4.90E-15
				Dicer_dimer	PF03368	634	722	7.80E-20
				Ribonuclease_3	PF00636	1034	1142	3.20E-20
				Ribonuclease_3	PF00636	1218	1365	3.50E-16

	Cryyphonectria parasitica	DCL1_CRYPA	Q2VF19	ResIII	PF04851	99	271	1.60E-12
				Helicase_C	PF00271	425	546	5.90E-17
				Dicer_dimer	PF03368	624	714	5.50E-18
				Ribonuclease_3	PF00636	1084	1196	8.00E-17
				Ribonuclease_3	PF00636	1285	1410	3.30E-18
		DCL2_CRYPA	Q2VF18	DEAD	PF00270	65	228	1.90E-18
				Helicase_C	PF00271	412	535	6.60E-16
				Dicer_dimer	PF03368	603	698	4.70E-23
				Ribonuclease_3	PF00636	1003	1111	1.90E-20
				Ribonuclease_3	PF00636	1189	1351	1.80E-13
	Aspergillus niger	DCL1_ASPNC	A2RAF3	ResIII	PF04851	119	287	5.40E-13
				Helicase_C	PF00271	456	563	4.40E-15
				Dicer_dimer	PF03368	641	727	5.90E-21
				Ribonuclease_3	PF00636	1080	1192	1.40E-20
				Ribonuclease_3	PF00636	1280	1393	1.20E-18
		DCL2_ASPNC	A2QX45	DEAD	PF00270	25	184	2.30E-18
				Helicase_C	PF00271	349	466	7.90E-16
				Dicer_dimer	PF03368	536	625	3.80E-20
				Ribonuclease_3	PF00636	928	1033	2.40E-13
				Ribonuclease_3	PF00636	1112	1257	7.50E-13
	Aspergillus oryzae	DCL1_ASPOR	Q2U6C4	ResIII	PF04851	117	286	1.20E-13
				Helicase_C	PF00271	451	561	7.80E-15
				Dicer_dimer	PF03368	640	726	8.90E-22
				Ribonuclease_3	PF00636	1078	1190	1.90E-18
				Ribonuclease_3	PF00636	1278	1391	4.30E-19
		DCL2_ASPOR	Q2UNX5	DEAD	PF00270	18	152	4.50E-16
				Helicase_C	PF00271	365	489	2.90E-15
				Dicer_dimer	PF03368	562	651	1.90E-23
				Ribonuclease_3	PF00636	947	1052	1.40E-20
				Ribonuclease_3	PF00636	1128	1275	3.20E-18

Basidiomycota	*Coprinopsis cinerea*	Hypothetical protein_COPC	CC1G_00230	Helicase_C	PF00271	385	486	1.80E-05
				Dicer_dimer	PF03368	578	668	2.40E-10
				PAZ	PF2170	818	956	2.70E-02
				Ribonuclease_3	PF00636	990	1166	2.20E-19
				Ribonuclease_3	PF00636	1245	1365	4.50E-17
		Hypothetical protein_COPC	CC1G_03181	DEAD	PF00270	37	170	1.30E-15
				Helicase_C	PF00271	423	536	5.30E-14
				Dicer_dimer	PF03368	613	733	2.30E-21
				Ribonuclease_3	PF00636	1509	1690	8.50E-17
				Ribonuclease_3	PF00636	1769	1930	5.70E-16
		TypeIII restriction enzyme_COPC	CC1G_13988	ResIII	PF04851	21	184	6.50E-15
				Helicase_C	PF00271	370	491	4.20E-15
				Dicer_dimer	PF03368	571	657	6.10E-16
				Ribonuclease_3	PF00636	975	1082	1.70E-23
				Ribonuclease_3	PF00636	1155	1267	5.80E-19
	Laccaria bicolor	Hypothetical protein_LACBS	382788	Dicer_dimer	PF03368	630	720	3.00E-11
				PAZ	PF2170	908	1032	3.70E-08
				Ribonuclease_3	PF00636	1071	1251	1.60E-20
				Ribonuclease_3	PF00636	1331	1450	3.50E-18
	Galerina marginata	Hypothetical protein_GALM	150895	Helicase_C	PF00271	445	555	0.00011
				Dicer_dimer	PF03368	645	735	9.40E-12
				PAZ	PF2170	925	1051	1.20E-07
				Ribonuclease_3	PF00636	1090	1320	6.40E-20
				Ribonuclease_3	PF00636	1399	1526	3.00E-15
